# Host-specific plasmid evolution explains the variable spread of clinical antibiotic-resistance plasmids

**DOI:** 10.1073/pnas.2212147120

**Published:** 2023-04-06

**Authors:** Fabienne Benz, Alex R. Hall

**Affiliations:** ^a^Department of Environmental Systems Science, Institute of Integrative Biology, ETH Zurich, Zurich 8092, Switzerland

**Keywords:** antibiotic resistance, plasmids, evolution

## Abstract

Antibiotic resistance encoded on plasmids is a pressing global health problem. Predicting which plasmids spread in the long term remains very challenging, even though some key parameters influencing plasmid stability have been identified, such as plasmid growth costs and horizontal transfer rates. Here, we show these parameters evolve in a strain-specific way among clinical plasmids and bacteria, and this occurs rapidly enough to alter the relative likelihoods of different bacterium–plasmid combinations spreading. We used experiments with *Escherichia coli* and antibiotic-resistance plasmids isolated from patients, paired with a mathematical model, to track long-term plasmid stability (beyond antibiotic exposure). Explaining variable stability across six bacterium–plasmid combinations required accounting for evolutionary changes in plasmid stability traits, whereas initial variation of these parameters was a relatively poor predictor of long-term outcomes. Evolutionary trajectories were specific to particular bacterium–plasmid combinations, as evidenced by genome sequencing and genetic manipulation. This revealed epistatic (here, strain-dependent) effects of key genetic changes affecting horizontal plasmid transfer. Several genetic changes involved mobile elements and pathogenicity islands. Rapid strain-specific evolution can thus outweigh ancestral phenotypes as a predictor of plasmid stability. Accounting for strain-specific plasmid evolution in natural populations could improve our ability to anticipate and manage successful bacterium–plasmid combinations.

## Significance

Antibiotic resistance is a major challenge in treating bacterial infections. Resistance genes are often on mobile genetic elements called plasmids. Being able to predict which bacterium–plasmid combinations are most successful, including in the absence of antibiotics, would help manage resistance. We used experiments with clinical bacteria to show key parameters affecting the spread of plasmids varied among different bacterium–plasmid combinations, but this was not sufficient to predict which combinations were most successful in the long term. Instead, accounting for rapid evolution of plasmids significantly advanced our ability to explain which combinations did best, because plasmid evolution depended critically on which bacterial host they were carried by. Accounting for rapid, strain-specific plasmid evolution may help predict and combat resistance.

Plasmids are self-replicating genetic entities that play a major role in bacterial ecology and evolution. They supply their bacterial host with functional innovations, such as antibiotic- and heavy metal resistance and virulence factors (1, 2). Particularly problematic in clinical contexts are conjugative plasmids carrying antibiotic-resistant genes, which can transfer horizontally (3, 4). To manage the dissemination of resistance plasmids and associated morbidity, mortality, and health-care costs, we need to understand the factors that drive their spread or decline in bacterial populations (5, 6). Some plasmids are associated with specific bacterial lineages, and these “successful” bacterium–plasmid combinations are a primary driver of the global dissemination of antibiotic resistance (6, 7). Yet it remains unclear why some combinations are more successful than others. For example, the high prevalence of *Escherichia coli* (*E. coli*) of sequence type 131 (ST131, clade C) with IncF-family plasmids encoding the extended spectrum beta lactamase (ESBL) *bla*_CTX-M_ has been ascribed to a competitive advantage during gut colonization (8–10), but it is not clear why this particular bacterium–plasmid combination, and not others, has achieved this ecological success. Despite this, some key parameters affecting plasmid ecological success have been identified (11). Here, we ask whether the variable spread/decline of different clinical bacterium–plasmid combinations in the absence of antibiotics can be explained by variation of these parameters, both among different combinations and over evolutionary time.

Plasmid persistence is generally expected to depend on the balance of growth costs (plasmid effects on bacterial replication), transfer rates (typically by conjugation) and, to a lesser extent given many natural plasmids carry addiction systems, segregational loss (12–16). Past work showed that these traits vary depending on the bacterium–plasmid combination (17–21). This variability may therefore be central to predicting long-term plasmid stability (over hundreds of generations and in the absence of antibiotics). However, bacteria and their plasmids can evolve rapidly, for example to reduce growth costs or alter transfer rates (22–31). Such evolutionary changes may reduce the predictive power of plasmid stability traits measured at a given point in time. In particular, if different bacterium–plasmid combinations vary in their propensity for evolutionary changes in plasmid stability traits, then initial variation of those traits may be a poor predictor of which combinations are most stable in the long term. Whether and how evolution of plasmid stability traits varies among bacterium–plasmid combinations remains poorly understood. Despite this, epistasis has been documented for chromosomal mutations affecting plasmids (24, 32), indicating their evolutionary trajectories may indeed be specific to individual combinations. In summary, we know plasmids and bacteria evolve rapidly, but is this fast, strong, and variable enough to “reshuffle the deck” and change which bacterium–plasmid combinations are most stable in the long term?

Here, we test whether the spread/decline of resistance plasmids in experimental populations of *E. coli* strains over 15 d (~150 generations) in the absence of antibiotics is explained by i) initial variation of plasmid stability traits among bacterium–plasmid combinations and/or ii) variable evolution of plasmid stability traits during the experiment. We generated multiple bacterium–plasmid (hereafter, strain–plasmid) combinations from clinical *E. coli* strains and their natively associated ESBL plasmids. This approach overcomes some limitations of previous research into the role of rapid evolution in the spread of plasmids, which has typically focused on one strain at a time (33, 34), indirect genomic evidence from bacteria evolving in clinics (35–37), or model strains/plasmids (16, 26). Using clinical strains/plasmids is particularly important here, first because they are more relevant for the resistance crisis, and second because genetic factors, such as other plasmids and virulence determinants that are lacking from laboratory strains, may play key roles in the spread of the focal resistance plasmids we aim to understand (38, 39). We tracked the frequencies of ESBL plasmid-carrying clones during serial passage in the absence of antibiotics and quantified plasmid stability traits of ancestral and evolved clones. Implementing these as parameters in a mathematical model, we simulated plasmid dynamics for each strain–plasmid pair. With analyses of whole-genome sequences, we identified parallel, yet strain–plasmid pair-dependent, evolution. Together, this shows how rapid plasmid evolution can override initial variation among strain–plasmid pairs and reveals its dependence on the specific strain–plasmid combination, as exemplified by epistatic plasmid mutations increasing plasmid transfer rates.

## Results

### Stability of Resistance Plasmids Varies among Strain–Plasmid Combinations.

**Fig. 1.**
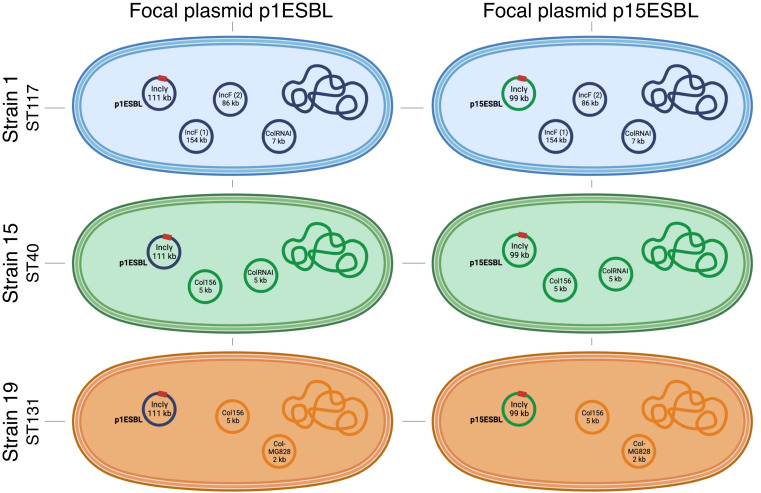
Six unique strain–plasmid pairs. Clinical *E. coli* strains (1, 15, and 19; rows) each carrying one of the two focal plasmids (p1ESBL, an ESBL plasmid originally from strain 1, or p15ESBL, an ESBL plasmid originally from strain 15; columns). The red square represents an ESBL-encoding gene on the focal plasmid in each strain; ST gives the sequence type of each strain. In cases where plasmids carry multiple replicons, only one is shown. See *SI Appendix*, Table S3 for detailed description of plasmids and their antibiotic-resistance genes.

**Fig. 2.**
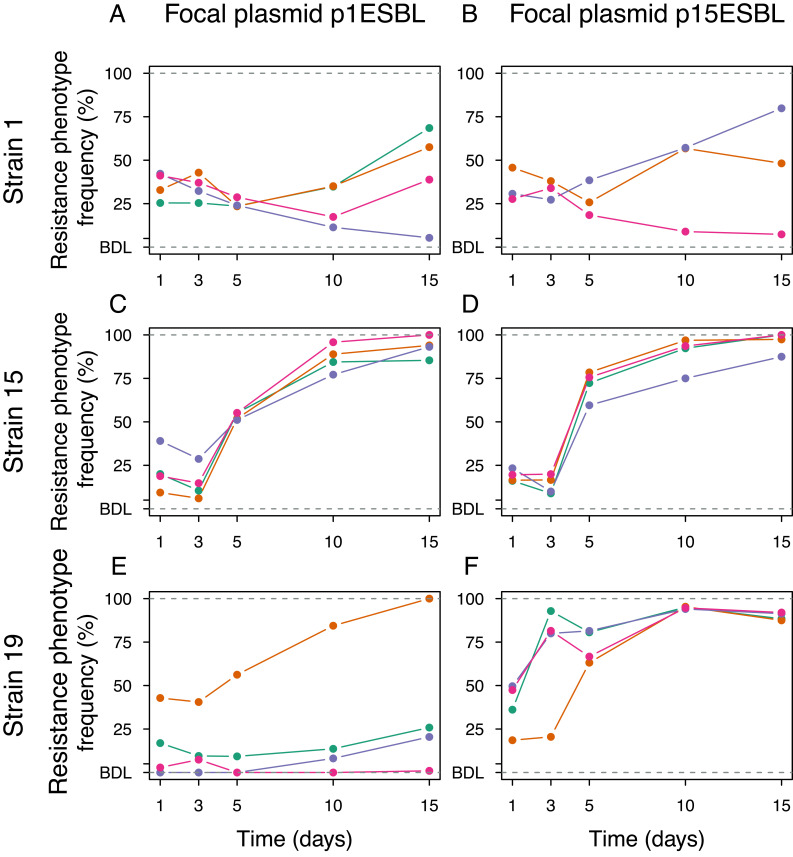
Strain–plasmid pair-dependent plasmid dynamics in the absence of antibiotics. Panels *A* to *F* show the different strain–plasmid pairs. In each panel, colors indicate the four replicate populations (three in *B*) and are maintained in the other figures. The *y* axis gives the fraction of colony-forming units with an ESBL phenotype (approximating the frequency of plasmid-carrying cells, which we confirmed by PCR; see *SI Appendix*, *Supplementary Methods*) in each replicate population sampled at days 1, 3, 5, 10, and 15. Points below the detection limit (BDL) indicate cases where no ESBL-positive cells were recovered during replica plating.

We generated six unique strain–plasmid pairs, each derived from one of the three *E. coli* strains isolated from hospital patients and one of the two ESBL-encoding clinical resistance plasmids natively associated with the same strains (Fig. 1). To monitor the spread/decline of each ESBL plasmid in populations of each strain, we made replicate microcosm cultures, each containing a mixture of a plasmid-carrying strain and the corresponding plasmid-free strain. We then serially passaged each population for 15 d in the absence of antibiotics. We used the frequency of resistance phenotypes as a proxy for the frequency of ESBL-plasmid carrying clones over time and verified this reflected plasmid carriage by PCR screening (*SI Appendix*, *Supplementary Methods* and Tables S1 and S2). In all populations, ESBL plasmids persisted over 15 d of antibiotic-free serial passage (Fig. 2). However, the temporal plasmid dynamics varied among strains and plasmids (two-way ANOVA, effect of plasmid: *F*_1,17_ = 14.758, *P* < 0.01; effect of strain: *F*_2,17_ = 7.834, *P* < 0.01; the response variable here is the area under the curve (AUC) of plasmid frequency over time, a measure of time-averaged plasmid success). The differences among plasmids also depended on which strain they were carried by (plasmid × strain interaction: *F*_2,17_ = 6.672, *P* < 0.01). For example, both plasmids spread rapidly in populations of strain 15, but showed different trajectories in populations of strain 19 (Fig. 2). This difference in long-term stability between the two plasmids is surprising given their high sequence similarity (*SI Appendix*, Fig. S1). One possible explanation would be an average difference in their initial frequencies in microcosms containing the different strains, but we excluded this in a further experiment (*SI Appendix*, Fig. S2). There was also marked variation among replicate populations in some combinations, such as those with strain 1 carrying either plasmid (Fig. 2).

### Variable Plasmid Dynamics Are Poorly Explained by Initial Variation of Plasmid Stability Traits.

**Fig. 3.**
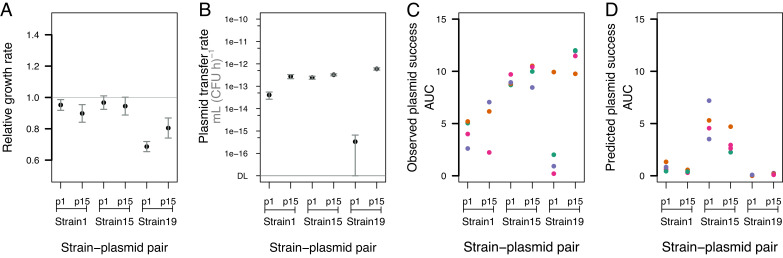
Ancestral plasmid stability traits explain only some of the observed plasmid dynamics. (*A*) Plasmid growth costs, expressed as population growth rate of plasmid-carrying relative to equivalent plasmid-free strains in the absence of antibiotics. (*B*) Plasmid transfer rates of each ancestral strain–plasmid pair, measured in mating assays with each corresponding plasmid-free equivalent as the recipient strain (*Material and Methods*). (*C*) Plasmid success, estimated as the AUC of plasmid frequency over time, for each strain–plasmid pair during the 15-d experiment shown in Fig. 2. (*D*) Plasmid success predicted by a model simulating plasmid frequencies over time, based on parameter values from panels *A* and *B* [hereafter, the ancestral (Anc) model], with $C_{0}=1.15\times 10^{12}$ (*Material and Methods*).

We estimated plasmid stability traits for each ancestral strain–plasmid pair (Fig. 3 *A* and *B*). Growth costs (population growth rate of a plasmid-carrying strain relative to its plasmid-free equivalent) varied depending on the bacterial strain, but not which of the two plasmids it carried (two-way ANOVA, effect of strain: *F*_2,26_ = 10.031, *P* < 0.001; effect of plasmid *F*_1,26_ = 0.114, *P* = 0.739; Fig. 3*A*), with particularly large costs for both plasmids with strain 19. Transfer rates varied depending on the plasmid, the host strain, and their interaction (two-way ANOVA, plasmid effect: *F*_1,12_ = 76.620, *P* < 0.001; strain effect *F*_2,12_ = 6.627, *P* < 0.05; plasmid × strain interaction *F*_2,12_ = 19.360, *P* < 0.001; Fig. 3*B*). For example, transfer rate of p1ESBL in strain 19 was very low compared to p15ESBL, whereas both plasmids transferred similarly well in strain 15. We did not detect significant plasmid loss for any strain–plasmid pair over 24 h (*SI Appendix*, Fig. S3), which we attribute to the toxin–antitoxin (TA) systems present on both plasmids (*Material and Methods*). Qualitatively, this variation in ancestral plasmid stability traits (Fig. 3 *A* and *B*) is consistent with some of the observed plasmid dynamics over 15 d (Figs. 2 and 3*C*): Both plasmids spread rapidly with strain 15 and these combinations had relatively low costs and high transfer rates. However, other long-term trends were not consistent with the ancestral variation of plasmid stability traits, such as the rapid spread of p15ESBL with strain 19 (Figs. 2 and 3*C*) despite this combination having the second largest fitness cost (Fig. 3*A*).

We next tested the predictive power of the ancestral plasmid stability traits quantitatively, using a variation of the well-established population dynamics model by Simonsen et al. (40), modified by Huisman et al. (41), to account for variable growth costs and transfer rates (*SI Appendix*, *Supplementary Model Information, sections I–IV* and Figs. S4–S8). Consistent with observed dynamics (Figs. 2 and 3*C*), this model (Anc Model) predicted both plasmids to spread effectively in populations of strain 15 (Fig. 3*D*). With strain 19, the model matched the observed decline of p1ESBL, but not the high frequency of p15ESBL (Fig. 3 *C* and *D*). At the level of individual replicates, which are accounted for in the model by including the initial subpopulation densities specific to each replicate, the model failed to predict observed variation among replicates of strain 1 with both plasmids and strain 19 with p1ESBL. An alternative version of the model that resulted in more realistic dynamics in terms of total population growth (but not plasmid dynamics) produced a very similar outcome (*SI Appendix*, *Supplementary Model Information, section III*). In summary, ancestral plasmid stability traits varied among strain–plasmid pairs, but this information explained only some of the observed plasmid dynamics.

### Phenotypic Evolution Varies among Strain–Plasmid Pairs and Explains Variable Plasmid Stability.

**Fig. 4.**
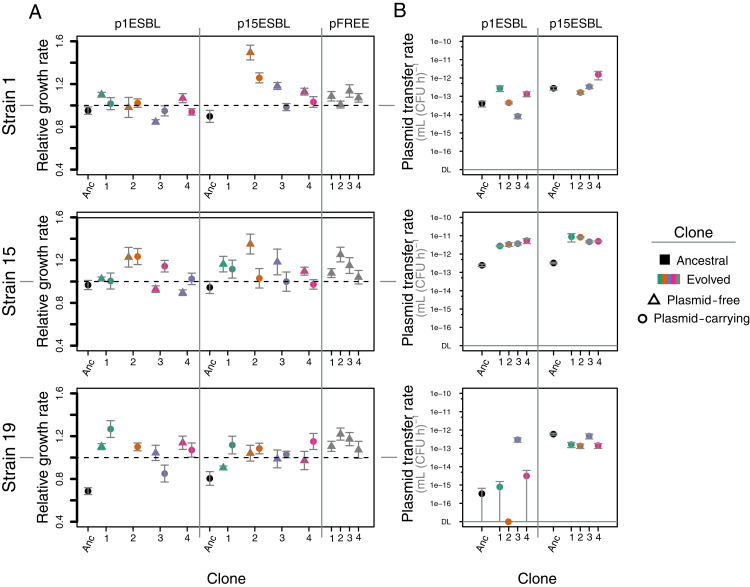
Strain–plasmid combination-dependent evolutionary changes. (*A*) Population growth rates for ancestral plasmid-carrying, evolved plasmid-carrying, and evolved plasmid-free clones (see legend/*x* axis) for each strain–plasmid combination, and for evolved clones from plasmid-free control cultures (pFREE at the *Right* of each panel). Growth rate in each case is shown relative to the corresponding plasmid-free ancestral strain. (*B*) Plasmid transfer rates for ancestral and evolved plasmid-carrying clones (see legend/*x* axis) in each strain–plasmid combination. Transfer rates were measured in mating assays with the corresponding ancestral plasmid-free strain as the recipient. For one replicate population with strain 19-p1ESBL (orange), we found no ampicillin-susceptible colonies at the end of the experiment, so only the plasmid-carrying evolved clone is shown.

To test for evolutionary changes in plasmid stability traits, we first measured population growth rates of evolved (day 15) and ancestral (day 0) ESBL plasmid-carrying (p^+^) and plasmid-free (p^−^) clones (Fig. 4*A*). For all strain–plasmid pairs, growth rates of evolved p^+^ clones were higher than those of the corresponding ancestral p^+^ clones. In some populations, evolved p^+^ clones also had higher growth rates than those of evolved p^−^ clones from the same population, but this varied among strain–plasmid combinations (ANOVA with growth rate of evolved p^+^ relative to evolved p as the response variable: plasmid × strain interaction: *F*_2,16_ = 6.453, *P* < 0.01). For example, evolved p15ESBL^+^ clones were consistently slower growing than evolved p^−^ clones from the same populations with strains 1 and 15, but not with strain 19. This indicates that growth costs of plasmids, both relative to ancestral clones and relative to coexisting plasmid-free clones, were reduced to different degrees depending on strain–plasmid combination. For p^−^ control populations, initiated with only plasmid-free versions of each ancestral strain (pFREE in Fig. 4*A*), evolved clones of all strains had higher growth rates than those of equivalent ancestral clones, showing that there was also adaptation to the experimental environment not linked to plasmid carriage.

To test for evolutionary changes in plasmid transfer rates, we performed mating assays between evolved/ancestral p^+^ clones and their ancestral p^−^ equivalents (Fig. 4*B*). This revealed strong strain dependence: Both plasmids increased their transfer rates in all replicate populations with strain 15 (~10- to 20-fold for p1ESBL and 20- to 35-fold for p15ESBL), but much less consistently and to a smaller extent with the other two strains (aligned rank transformation ANOVA with transfer rate of evolved clones relative to ancestral as response variable, strain effect: *F*_2,16_ = 6.2942, *P* < 0.01). Changes in transfer rate also depended on the plasmid itself and the strain–plasmid combination (plasmid effect: *F*_1,16_ = 8.4926, *P* = 0.01; plasmid × strain interaction: *F*_2,16_ = 3.7065, *P* < 0.05). We found no evidence that variation of plasmid success over 15 d was linked to variable segregational plasmid loss among different evolved clones within each strain–plasmid combination or on average among the different combinations (measured over 24 h; *SI Appendix*, Fig. S9). Thus, both growth costs and transfer rates changed during our experiment, in ways dependent on the strain–plasmid combination.

**Fig. 5.**
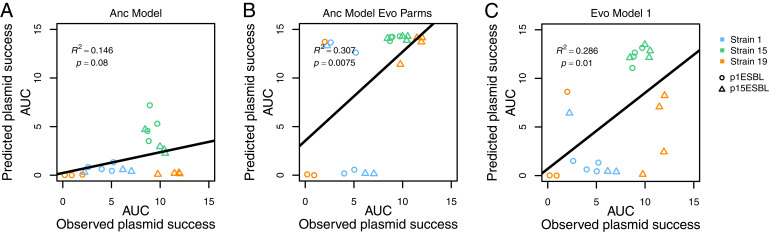
Evolutionary changes in plasmid stability traits explain long-term plasmid stability better than ancestral values do. Plasmid success over time (AUC) observed in our 15-d serial passage experiment (*x* axis in each panel) is plotted against values predicted by three different versions of the population dynamic model (panels *A*–*C*), for each replicate of each strain–plasmid combination (see legend). (*A*) Ancestral model accounting for ancestral plasmid stability traits only (as in Fig. 3*D*). (*B*) Ancestral model implemented with evolved parameters, (*C*) Evolved model accounting for plasmid stability traits of ancestral and evolved clones. See *SI Appendix*, *Supplementary Model Information* for model details. Note replicate population 3/orange of strain 19-plasmid 1 is excluded in model comparisons because no susceptible evolved clones were isolated from that population (*Material and Methods*).

We tested whether the phenotypic changes specific to strain–plasmid combinations observed above explained the variable long-term stability of plasmids (Fig. 2). We used the same population dynamics model as above (Anc Model), but with parameters from evolved strains (*SI Appendix*, *Supplementary Model Information, section I*). This updated model (Anc Model Evo Parms) explained more of the experimentally observed variation of plasmid stability than the Anc Model did (simulated vs observed plasmid success: *R*^2^ = 0.31, *P* < 0.05; Fig. 5 *A *and *B*). The two model versions even predicted different rank orders in terms of which strain–plasmid combinations would be most successful. For example, the version using evolved parameter estimates predicted p15ESBL to spread more successfully with strain 19 than the version based on ancestral parameters did (Fig. 5 *A* and *B*), and this was much closer to what we observed in our 15-d experiment. We also tested the predictive power of more complex model versions accounting for evolutionary change (*SI Appendix*, *Supplementary Model Information, section II*), simulating a greater number of evolved bacterium–plasmid combinations and more realistic starting conditions (with mutants initially present at very low frequency). As for Anc Model Evo Parms, these models had greater explanatory power (*R*^2^ = 0.29, *P* < 0.05 for Evo Model 1; Fig. 5*C* and *SI Appendix*, Fig. S7) than that of the Anc Model, confirming that accounting for evolutionary changes in plasmid transfer rate and growth costs improved our ability to explain variable long-term plasmid stability. Together, these results suggest that rapid evolution of plasmid stability traits can change which strain-plasmid combinations are most successful, thereby “reshuffling the deck” and manifesting the role of evolution in identifying successful strain–plasmid pairs.

### Parallel Genetic Changes in ESBL Plasmids and Chromosomes Are Specific to the Strain–Plasmid Combination.

**Fig. 6.**
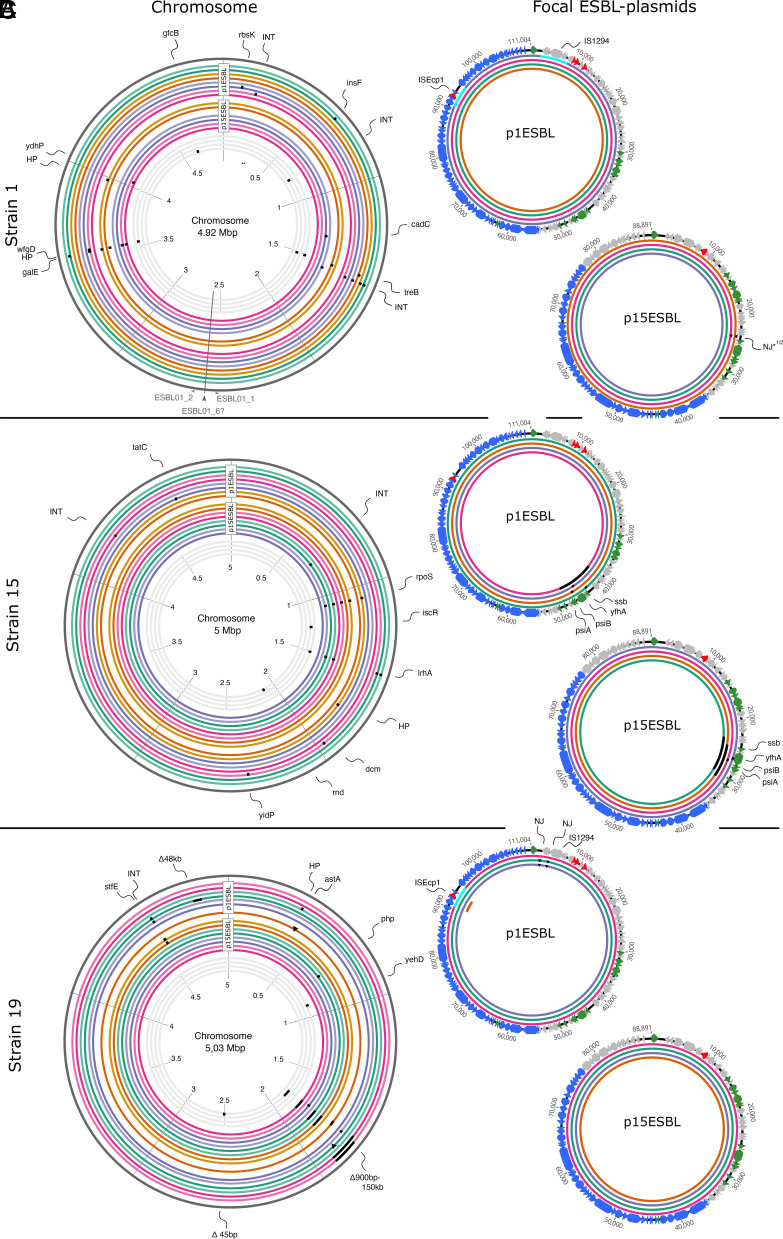
Genetic changes in chromosomes and ESBL plasmids after 15 d of serial passage without antibiotics. Chromosomes (*Left*) are shown for each strain (*A*–*C*) evolved with either of the two focal ESBL plasmids (shown at *Right*). In chromosome plots, the eight outermost circles are clones isolated from populations evolved with p1ESBL, the eight middle circles are clones isolated from populations with p15ESBL, and the four innermost circles are clones from plasmid-free control populations. Colors indicate replicate populations; for each population, two evolved clones are shown (darker shade = plasmid carrying; lighter shade = plasmid free). Black squares show nonsynonymous genetic changes and arrows indicate target genes/regions. In the chromosome, only deletions of 20 kb and larger are to scale, triangles indicate new junctions (NJ), and turquoise squares indicate inversions of that region. In the plasmid plots, colored rings identify replicate populations (as in chromosome plots). Plasmid genes are colored according to predicted function: red = antibiotic resistance, blue = transfer genes, green = stability genes, gray = other. Nonfocal plasmids did not show genetic changes and are not depicted. Dataset S6 gives a detailed list of genetic changes.

We whole-genome sequenced ancestral strain–plasmid combinations [short- and long read-sequencing (38, 42)] and single end point clones (p^+^/p^−^, Illumina only) from each replicate population. First, we found that each evolved clone maintained all their native non-ESBL plasmids. Thus, we could exclude changes in carriage of nonfocal plasmids as a driver of observed variation in ESBL plasmid dynamics (Figs. 2 and 3*C*). Further, we found that plasmid ColRNAI from strain 1 had cotransferred to strain 19 when generating strain19-p1ESBL, where it was maintained in two out of the 4 sequenced ESBL plasmid-carrying evolved clones (green and orange). Sequence data also revealed strain-specific parallel evolution (Fig. 6). In combination with strain 15, both ESBL plasmids showed parallel genetic changes in every independently evolved replicate clone (same plasmid region, different nucleotide changes; Fig. 6*B*). These genetic changes on plasmids were specific to strain 15 (not found in other evolved clones; Fig. 6) and coincided with increased plasmid transfer rates in these clones (Fig. 4*B*).

In several cases, we detected genetic changes involving interactions between loci on ESBL plasmids and other mobile genetic elements, including mobile elements encoded on the ESBL plasmids themselves (Datasets S1 and S2). For example, in the ancestral p1ESBL, the shufflon segment B is disrupted by a 2,880 bp transposition unit, encoding the insertion sequences ISEcp1 and the *bla*_CTX-M-1_ gene (*SI Appendix*, Fig. S11). In one sequenced clone each of strain 19 and strain 1, the shufflon segments of p1ESBL rearranged such that the entire transposition unit was inverted (Fig. 6 *A* and *C*, turquoise squares). In these two populations, p1ESBL was at very low frequency at day 15 (Fig. 2) and showed decreased transfer rates in combination with strain 1 (Fig. 4*B*). In a strain 19 clone from another replicate population, the same mobile element jumped to the chromosome (Fig. 6*C* (*astA*) and *SI Appendix*, Fig. S11), while the same clone lost p1ESBL, explaining the nontransferable resistance phenotype of this evolved clone (Fig. 4*B*). The other plasmid, p15ESBL, also showed evidence of interactions among genetic elements: p15ESBL from two strain 1 clones from different replicate populations had a new genetic junction (NJ, triangles in Fig. 6; here NJ*^1/2^ in Fig. 6*A*) with a putative group II intron. This intron is also present in the ancestral IncF plasmid and p1ESBL of strain 1 (ESBL01_04913, Dataset S3). Furthermore, this region in p1ESBL is identical to the position of NJ*^1/2^ in p15ESBL and is adjacent to the mutated locus on ESBL plasmids with strain 15 described above (Fig. 6*B*). Thus, several genetic changes and interactions with other mobile elements occurred in this region of our plasmids. Finally, we detected evidence of moderately increased copy number for evolved vs ancestral plasmids, with one strain 19-plasmid 1 replicate showing a particularly large increase (*SI Appendix*, Fig. S10).

Genetic changes in bacterial chromosomes also demonstrated a high level of parallelism and, as above for plasmid mutations, often involved accessory genomic features specific to clinical or pathogenic bacteria, such as pathogenicity islands (PAIs). Each sequenced strain 19-p15ESBL clone (from independent populations) had a 900 bp −150 kb deletion of or within a putative chromosomal PAI (Fig. 6*C* and Dataset S4). These deletions were specific to p^+^ clones, which consistently had a growth advantage over the corresponding p^−^ end point clones (Fig. 4*A*). Also in strain 1, ~20 kb of a putative chromosomal PAI (*SI Appendix*, Fig. S12 and Dataset S5) was deleted in 8 out of the 19 sequenced evolved clones, but never in clones carrying p15ESBL. We found parallel mutational changes in *rpoS* (central regulator of the general stress response) specific to p^−^ end point clones only in populations of strain 15 with p15ESBL, and in one case with p1ESBL. These mutations were either frameshift mutations or SNPs introducing a preliminary stop codon, and all these evolved p^−^ clones could outgrow the evolved p15ESBL-carrying clones from the same populations (Fig. 4*A*). In summary, chromosomal evolution depended on both the host strain and which ESBL plasmid was present, and using clinical strains here allowed us to detect adaptive changes in genetic elements such as PAIs that are often not represented in laboratory or model strains.

### Epistatic Changes in Plasmid Leading Region Explain Strain-Specific Increases in Transfer Rates.

**Fig. 7.**
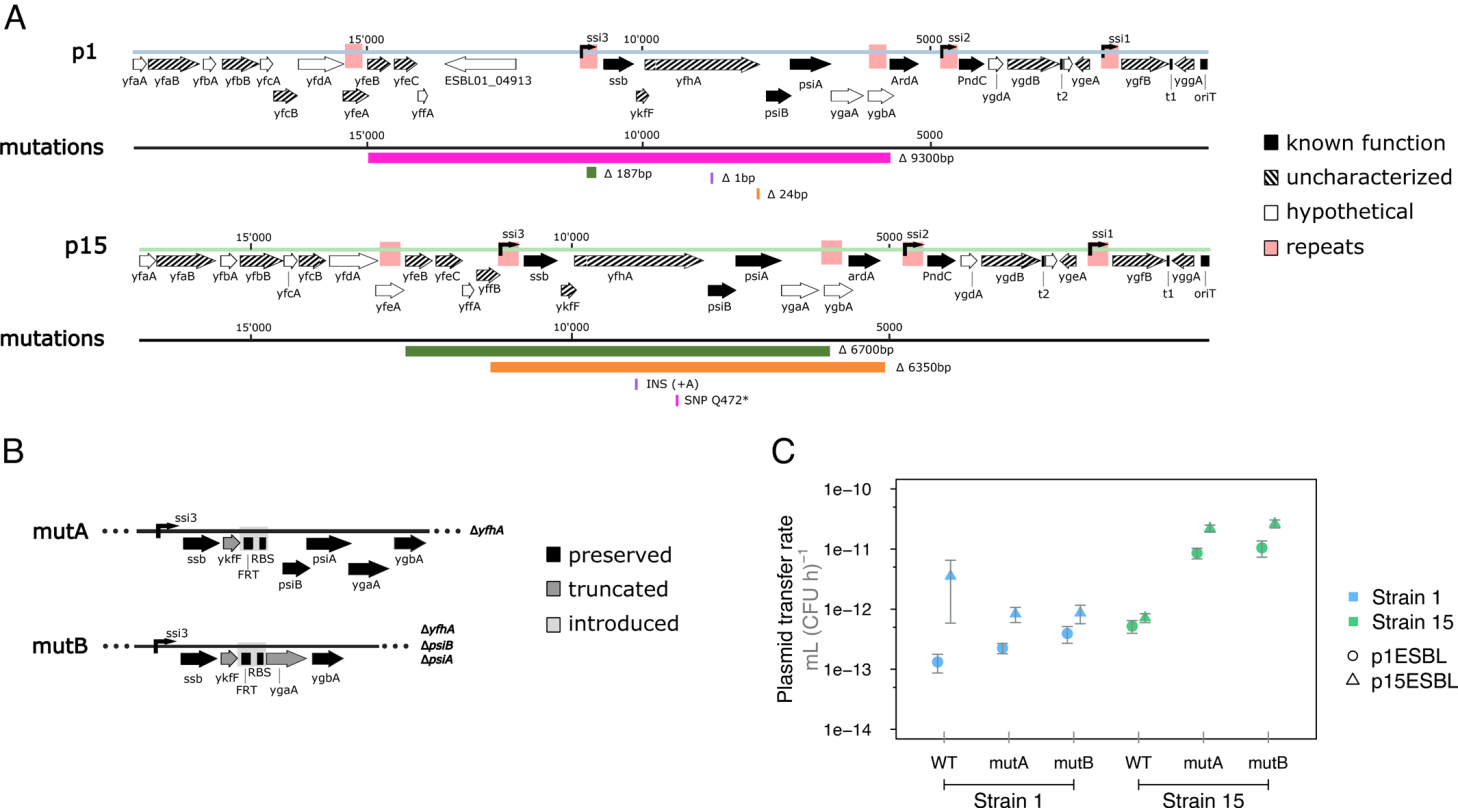
Genetic changes in the plasmid leading region increase transfer rate in a host-specific way. (*A*) Plasmid leading region (*oriT* to ~12 kb downstream) of p1ESBL and p15ESBL and mutations accumulated during the evolution experiment. This region consists of three operons, transcribed by *ssi3-1*, encoding the following gene products: SSB (ssDNA binding protein), YfhA (unknown function), PsiB-PsiA (SOS-response inhibition), ArdA (antirestriction protein), PndC (postsegregation killing protein), and several open reading frames of unknown function. Further information about genes/functions is in Dataset S3. Mutation colors correspond to the replicate clone they were found in. (*B*) Schematic of the *ssi3* operon for the knockout mutants mutA and mutB. RBS is ribosomal binding site and FRT is flippase recognition target, introduced during mutant construction. Both mutants have a truncated *ykfF*. (*C*) Both mutant plasmids increased transfer rates with strain 15 only.

Having found above that evolved p1ESBL and p15ESBL in strain 15 increased their transfer rates, and acquired parallel genetic changes, we asked why these mutations were not observed in the other strains. These genetic changes were in the plasmid leading region, the first part of a conjugated plasmid to enter the recipient cell, and expressed immediately thereafter in single-stranded form via single-strand initiation promoters [*ssi,* (43, 44)]. Observed changes were likely loss-of-function mutations: They included SNPs introducing a preliminary stop codon, insertions leading to a frame shift, deletions within one operon or its promoter *ssi3*, or the entire operon (Fig. 7*A*). To test whether genetic changes in this region confer altered plasmid transfer rates, we generated two different knockout mutants disrupting the *ssi3* operon; mutA (Δ*yfhA*) and mutB (Δ*yfhA*, Δ*psiB,* Δ*psiA*) in both ancestral plasmids (p1ESBL and p15ESBL; Fig. 7*B*). In combination with strain 15, mutA and mutB both increased the transfer rates of both ESBL plasmids (Fig. 7*C*), suggesting that this region does indeed affect transfer rate. However, this was not the case with strain 1, demonstrating the effect of these mutations is strain specific (Fig. 7*C*; Welch two sample *t* test for strain 15, *P* < 0.05 in all cases before and after Holm’s correction for multiple testing; Wilcoxon rank-sum test for strain 1, *P* > 0.05 in all cases before and after Holm’s correction for multiple testing). Introducing mutA or mutB did not affect plasmid growth cost in strain 1 or strain 15 (*SI Appendix*, Fig. S13). Strain 19 is not included here because we could not reintroduce mutant plasmids into the ancestral plasmid-free strain 19 (*Material and Methods*). In summary, this plasmid locus, showing parallel strain-specific evolution in our 15-d experiment and coinciding with altered transfer rates, affected plasmid transfer in a strain-specific way. This is consistent with a strain-specific benefit of these plasmid mutations in terms of boosting transfer, and therefore plasmid abundance, in the absence of antibiotics. Moreover, that the same region was mutated in both plasmids suggests that mutations in the *ssi3* operon may be a general mechanism of adaptation in clinically relevant plasmids such as these.

## Discussion

To manage the global spread of antibiotic resistance, we need to understand which bacterium–plasmid combinations are most successful. Plasmid persistence in the absence of antibiotics has been shown to depend on plasmid stability traits (growth cost, horizontal transfer, and segregational loss), which vary among different bacterium–plasmid combinations (17, 19, 33, 38, 45). Our results show that evolutionary changes of these parameters can outweigh their initial variation among strain–plasmid combinations in explaining plasmid persistence, rapidly altering both absolute and relative stability across different combinations. For example, the strain–plasmid combination with the second-largest initial growth cost in our experiment was one of the most successful in the long term (strain 19-p15ESBL). Using a quantitative model to predict the relative stabilities of different strain–plasmid combinations, we gained more explanatory power (both quantitatively and qualitatively, in terms of the rank order of our predictions) by accounting for evolutionary changes occurring over 15 d. Strain-specific evolutionary changes included mutations affecting plasmid transfer rate, which we observed consistently with one of our strains but not with others. Genetic manipulation experiments demonstrated that mutations in the plasmid leading region have epistatic (here, strain-specific) effects on transfer rate. Thus, rapid evolution can reshuffle the deck and here played a greater role than initial variation among strain–plasmid combinations in directing the trajectories of clinically important plasmids in the absence of antibiotics.

Our finding of a strong role for rapid evolution here is particularly relevant for predicting the spread of resistance because we used clinical strains and plasmids (7). Clinical bacteria naturally carry other plasmids, accessory regions such as PAIs, phages (*SI Appendix*, Table S4), and other mobile elements (1, 46). We found such genetic elements play a key role in plasmid evolution: The genetic changes we detected often involved interactions within and among mobile elements, such as intron and transposon movements and loss of PAIs (Fig. 6 and *SI Appendix*, Fig. S12). We expect similar types of genetic changes to affect plasmid evolution in other settings for five main reasons. First, recent experimental evolution studies support the general principle that resident mobile genetic elements, such as insertion sequences, play a key role in the evolution of new bacterium–plasmid combinations (22, 47, 48). Second, movement and deletion of PAIs has been observed during evolution of other strains and species (49). Third, genetic changes occurred repeatedly at the same locus in two different plasmids in our experiment, showing that this region (the plasmid leading region) is a target for plasmid stability evolution in multiple plasmids. Fourth, the leading region is conserved; for example, the *ssi3* operon is very common in *E. coli* plasmids (64% of 420 *ssb*-encoding plasmids) (50). Fifth, genetic changes in this region have previously been described in bioinformatic analyses comparing plasmids of pathogenic *E. coli* isolated from human and cows (51), and in experimental evolution with other plasmids (30). Our dataset nevertheless has some limitations. For example, our combinatorial approach allowed us to test directly for strain-specific evolution, but this required using a limited number of strains and plasmids.

Our results provide insights into genetic mechanisms stabilizing plasmids during evolution. The plasmid leading region, where we observed parallel evolution of both ESBL plasmids, is known to play a role in plasmid establishment (52, 53). Our results show that changes here can also affect transfer rate evolution. Because the leading region is expressed transiently in recipient cells after transfer (54, 55), this indicates an important role for this stage of the plasmid lifecycle in explaining variability of overall transfer efficiency. Within the leading region, we consistently found changes affecting *yfhA* (Fig. 7*A*), and further experiments supported the relevance of this gene for plasmid transfer (Fig. 7*C*). Like several other gene families in the leading region (56), the function of the *yfhA* gene product is unknown. Structural prediction by Phyre^2^ (57) suggests homology between part of the *yfhA* product (26% coverage) and DNA partitioning proteins Spo0j/ParB, including a ParB/sulfiredoxin N-terminal-like domain and a central DNA-binding domain (Dataset S7). Given this putative DNA-binding activity, and *yfhA*’s location in the leading region, we hypothesize that it modulates transfer indirectly by influencing plasmid establishment in recipient cells (54, 55). This may involve plasmid-induced changes in recipient gene regulation, documented in other species including some conditionally altering transfer rate (58, 59). A second, related way *yfhA* could influence plasmid establishment is via regulation of the recipient’s SOS response. Such effects are known for other leading region proteins (44, 55, 60). This is also in line with recent evidence that many leading region genes, including a *yfhA* homolog, encode antidefense-related functions (56). Consistent with a possible role for SOS in plasmid uptake, and similar to other recent work (48), we found disruptive mutations in *rpoS* in p^−^ clones in some combinations (Fig. 6*B*; strain 15 p1ESBL/p15ESBL), although the detailed mechanism linking *rpoS* to plasmid carriage here is not known. Similarly, it is not clear why loss-of-function changes in *yfhA* increase, rather than decrease, transfer. Recent work deleting the *ssi3*-equivalent F*rpo2* promoter in IncF plasmids, silencing the *ssi3*-homolog F*rpo2* operon (61), showed no effect on plasmid transfer rates (54). Our results with strain 1 show a similar picture, but with strain 15 that deletion here can increase transferability. We speculate a possible contributor to this is specific genetic conflict with other loci in recipient cells (33), which we discuss further below. Thus, our results implicate the *ssi3* operon in the plasmid leading region, and *yfhA* in particular, as a target for evolutionary changes affecting plasmid transfer rate.

Another key implication of our results is that evolutionary stabilization of plasmids is host specific, demonstrated by the increased transfer rates we observed only with strain 15. The epistatic effects upon deletion in the *ssi3* operon in p1ESBL or p15ESBL (Fig. 7*C*) help to explain this strain specificity. This is also consistent with past work showing that the expression of *psiB* in the *ssi3* operon varies among host strains (55, 62). A possible molecular driver of strain-specific effects is interaction between plasmids (63). Our data are consistent with such interactions, in that strain 1 carries an IncF plasmid which also encodes the *ssi3* operon (*SI Appendix*, Fig. S14). Expression of the IncF-encoded *ssi3* operon could potentially mask the effects of *ssi3* mutations in incoming ESBL plasmids (Figs. 4*B* and 7*C*). A constraint here is that leading region expression requires the single-stranded form, and thus transfer of the IncF plasmid. Given this IncF plasmid carries transfer genes, but not the *traS* gene which would inhibit transfer to cells already carrying the same plasmid (64), we consider parallel transfer of both plasmids (ESBL/IncIγ and IncF) into the same recipient cells to be plausible. Unraveling the extent of such cotransfer and interactions among clinical plasmids is an open area for future work. Interactions are also possible between loci on the same plasmid or the same operon. This has been suggested for regulators encoded in the leading region of IncW plasmids (65). A further experiment provided some evidence of this: The effects of *in trans* expression of *yfhA* in recipient cells differed between wild-type and knockout plasmids (*SI Appendix*, Fig. S15), likely due to interaction with *ykfF* encoding a putative RNA-binding-like domain (Dataset S3). In summary, our results show that leading region mutations can drive transfer rate evolution, but this depends critically on the identity of the host strain and its plasmids. This supports the general principle that mutations affecting plasmid stability are contingent on the bacterium–plasmid combination, in line with recent work in other species (33).

In conclusion, our results show that rapid, strain-specific evolution of plasmid stability traits is a key predictor of which bacterium–plasmid combinations are most successful in the long term (over >100 generations and without antibiotics). Our findings address and go beyond the recent proposal to take compensatory evolution into account for such predictions (7), by showing that rapid evolution of transfer rates as well as growth costs should be accounted for here. This points to some key pathways for improved prediction, and ultimately management, of problematic plasmid–bacterium combinations. First, to further characterize the molecular signatures of successful combinations, such as the genetic changes and loci driving high transfer rates in our experiments, evolve-and-resequence experiments (66) could be combined with analyses of phenotypic and genotypic changes for a wider range of strains and plasmids. Recent experiments with a multidrug resistance plasmid in various *E. coli* strains showed that this approach can reveal both conserved and strain-specific evolutionary responses (48). Second, because many of the genetic changes driving our results were in loci specific to clinical and natural strains (e.g., PAIs and mobile elements), there is a clear increase in the value of such data when they come from the most relevant clinical strains/plasmids. A recent analysis of circulating plasmid variants and longitudinal samples from individual patients has shown that there is genetic variation and evolution in these settings affecting plasmid stability traits as we studied above, including transfer rates (67). Third, although plasmid stability traits measured in vitro can correlate well with in vivo properties (38), there are key differences such as spatial structure, the role of the host immune system, and the resident microbiota. Therefore, downstream applications of candidate molecular signatures and rapid, strain-specific evolution will benefit from validation in in vivo infection models.

## Material and Methods

### Bacterial Strains and Growth Conditions.

Strains 1, 15, and 19 were sampled from different patients in a transmission study at the University Hospital Basel, Switzerland. We previously described phenotypes and genotypes of ancestral strains 1 (ST117, GCA_016433325.1) and 15 (ST40, GCA_008370755.1) and the genome sequence of the ancestral strain 19 (ST131, commonly associated with ESBL plasmids, GCA_020907505.1) (38, 68, 69). These strains are natively associated with conjugative ESBL plasmids of the type IncIγ, nonconjugative IncF for strain 19, and carry a range of other (nonfocal, non-ESBL) plasmids (*SI Appendix*, Table S3). Focal ESBL plasmids all encode at least one TA system (pndA/B, and RHH-RelE for p1ESBL and pndA/B only for p15ESBL). We generated strain–plasmid pairs as shown in Fig. 1 by curing the native ESBL plasmid from each strain and reintroducing p1ESBL and p15ESBL (*SI Appendix*, *Supplementary Methods*). To test the effects of alterations in the *ssi3* operon of the plasmid leading regions, we constructed two knockout mutants (see *SI Appendix*, *Supplementary Methods*, also for the *yfhA* expression vector used in a separate complementation assay). Unless stated otherwise, we grew bacterial cultures at 37 °C and under agitation (180 rpm) in lysogenic broth (LB) medium, supplemented with appropriate amounts of antibiotics (none, 100 µg/mL ampicillin (Amp), 50 µg/mL kanamycin (Kan), 25 µg/mL chloramphenicol (Cm)). We performed experiments with four to six biological replicates, with the exception of conjugation assays to estimate plasmid transfer rates (n = 3). Culture volumes were 5 mL for culture tubes and 150 µL for 96-well plates. We stored isolates in 25% glycerol at −80 °C.

### Experimental Evolution.

We serially passaged bacterial cultures (1:1,000 dilution into 5 mL fresh LB every day for 15 d; ~150 generations in total) without antibiotics. To generate the starting populations, we grew ESBL plasmid-carrying and ESBL plasmid-free strains from independent single colonies overnight and with the appropriate antibiotics. From these grown cultures, we transferred 2.5 µL ESBL plasmid-carrying (p^+^) and ESBL plasmid-free (p^−^) cultures to their assigned tube. We mixed p^+^ and p^−^ cells at the start, rather than taking pure p^+^ cultures, so we could detect both net positive and net negative changes in frequency. On days 1, 3, 5, 10, and 15, we froze each bacterial population for long-term storage and plated them on LB plates in dilutions appropriate for replicate plating on Amp plates (quantitative detection limit of ESBL-carrying clones:plasmid-free clones at ~1:100) and undiluted directly on Amp plates to verify presence/absence of Amp-resistant phenotypes. After 15 d, we additionally tested each replicate population for strain genotype and ESBL plasmid presence by PCR. All except for one resistant (and thus potentially p^+^) clone tested positive for the ESBL plasmid by PCR (3 out of 72 tested colonies, see *SI Appendix*, *Supplementary Methods*). We therefore take resistance phenotype as a proxy for ESBL plasmid presence. In addition to the four replicate populations per strain–plasmid pair, we passaged four ESBL plasmid-free control populations for each strain. After 15 d of serial passage, we isolated two single clones, one p^+^ and one p^−^, from each end point population for phenotypic testing and whole-genome sequencing. One replicate population of strain1-p15ESBL (green) was contaminated (*SI Appendix*, *Supplementary Methods*) and thus excluded.

### Measuring Plasmid Stability Traits.

To test for a growth cost associated with ESBL plasmid carriage in the absence of antibiotics, we measured the population growth rates $\left(\psi \right)$ of plasmid-carrying and the corresponding plasmid-free clones in monocultures. We grew bacterial cultures in 96-well plates overnight, removed Amp from cultures of ESBL plasmid-carrying clones by pelleting all cultures with centrifugation and subsequent resuspension, and transferred ~1 µL to a plate containing fresh LB without antibiotics with a pin replicator. These cultures grew for 24 h without agitation, and we estimated growth rates (h^−1^) based on eleven manual optical density (OD) measurements (Tecan NanoQuant Infinite M200 Pro) using the R package Growthcurver (70). To calculate the growth rates of p^+^ clones relative to p^−^ equivalents, we divided the growth rate of each p^+^ replicate by the averaged growth rate of the corresponding p^−^ clone.

To estimate ESBL plasmid transfer rates γ , [mL (CFU h)^−1^], we used the Approximate Extended Simonsen Model, accounting for varying growth rates of donor, recipient, and transconjugants and estimating a time window for reliable estimation of γ (41). ESBL plasmids were always transferred to their ancestral plasmid-free equivalent marked with the Kan-resistance plasmid pBGS18 (38, 71). In brief, we grew independent overnight cultures of donor and recipient strains in the appropriate antibiotics, washed them by pelleting and resuspending, and added ~1 µL of 6.5-fold diluted donor and recipient cultures into 150 µL fresh LB with a pin replicator (total ~1,000-fold dilution). Mating populations grew for 6 h (Figs. 3*B*, 4*B*, and 7*C*; 5 h for *SI Appendix*, Fig. S15) without shaking, which was well within the estimated critical time window to avoid results being influenced by substantial ESBL plasmid transfer from emerging transconjugants (41). To enumerate the final cell densities, we plated the mating cultures at the end of the conjugation assays on selective LB plates, with a detection limit of ~ 20 CFU/mL, corresponding to a single transconjugant colony. For transfer rate estimates in Fig. 4*B*, we used corresponding growth rate estimates from Fig. 4*A*. For the other transfer rate estimates (Fig. 7*C* and *SI Appendix*, Fig. S15), we estimated growth rates by growing donor and recipient replicate populations as initially 1,000-fold diluted, antibiotic-free monocultures in the plate reader for 24 h, with hourly OD measurements. Because donor and recipient strains were of the same type, i.e., strains 1, 15, or 19, we assumed transconjugants to grow at equal rates to the corresponding donor strains. Finally, we estimated γ with the R-package conjugator (41). We performed the transfer assay with complementation of *yfhA* in recipient strains (*SI Appendix*, Fig. S15) as described above with some alterations: independent overnight cultures grew with 0.2% glucose to repress promoter pBad. We washed them twice by pelleting and resuspending and subcultured them for 1.5 h in 2% L-arabinose to induce the expression of *yfhA*. We washed cultures to remove antibiotics and adjusted them to OD (600 nm) = 0.5 and mixed equal volumes of donor and recipient cultures to initiate the transfer assay.

We tested for evidence of plasmid instability due to segregational loss by measuring the change in the frequency of plasmid-carrying cells during overnight culture in the absence of antibiotics, starting with pure cultures of plasmid-carrying strains. Prior to the assay, we grew ESBL plasmid-carrying strains as independent overnight cultures with Amp and washed them by pelleting and resuspending. Prior to their pin replication into a 96-well plate containing fresh LB without antibiotics, we diluted and plated cultures on LB plates (t = 0 h). After 24 h of growth, we plated grown cultures on LB plates and replica-plated LB plates (t = 0 h and t = 24 h, respectively) on Amp plates to estimate the fraction of plasmid-carrying cells. We performed these assays for a subset of strain–plasmid pairs, including all ancestral combinations and evolved combinations that showed either low final frequencies or high among-replicate variation in the main experiment. Note that plasmid frequency in these assays can reflect a balance of segregational loss plus horizontal reacquisition and variable growth rates. Nevertheless, any drop in plasmid frequency after starting with a pure p^+^ culture indicates that individual cells can transition from p^+^ to p^−^.

### Population Dynamics Model.

We used a deterministic mathematical model, based on the model described by Simonsen et al. (40) and modified by Huisman et al. (41), to simulate plasmid dynamics in each strain–plasmid combination. More detailed information about the model can be found in *SI Appendix*, *Supplementary Model Information*. These models assume plasmids to transfer by mass action kinetics (in our experiments, bacterial populations grew in a well-mixed environment: liquid batch cultures with shaking). We modified the Extended Simonsen Model (41) by tracking only the dynamics of plasmid-carrying $\left(N_{P}\right)$ and plasmid-free $\left(N_{\mathrm{Ø Ø}}\right)$ cells, rather than donors, recipients, and transconjugants as in the original model. The differential Eqs. **1**–**3** describe the dynamics for these subpopulations ( $dN_{\mathrm{Ø Ø}}$ and  $dN_{P}$ ) and nutrient availability (d*C*) over time:

[1] $$\frac{dN_{Ø}}{dt}=\;\psi_{Ø}\frac{C}{q+C}N_{Ø}-\gamma \frac{C}{q+C}N_{P}N_{Ø},$$

[2] $$\frac{dN_{P}}{dt}=\;\psi_{P}\frac{C}{q+C}N_{P}+\gamma \frac{C}{q+C}N_{P}N_{Ø,}$$

[3] $$\frac{dC}{dt}={-(\psi_{Ø}N_{Ø}+\psi }_{P}N_{P})\frac{C}{q+C}e,$$

where cells grow at the maximal growth rate ψ (h^−1^) and plasmids are transferred at the maximal transfer rate γ [mL (CFU h)^−1^] from a plasmid-carrying to a plasmid-free bacterium. Both terms are regulated by the Monod function Cq+C , where *C* is the resource concentration (µg mL^−1^) and *q* the half-saturation constant (µg mL^−1^); *e* represents a conversion factor of resources into bacterial cells. We also used an expanded version of the model, accounting for evolutionary changes in plasmid stability traits by including subpopulations of ancestral/evolved bacteria and plasmids in various combinations, incorporating experimentally determined estimates of both ancestral and evolved plasmid stability traits (Evo Model 1; equations 4 to 10 in *SI Appendix*, *Supplementary Model Information, section II*).

To simulate the expected change in plasmid frequency over time in each replicate and each strain–plasmid combination, we used experimentally determined values of ψ and γ for each ancestral (prior to experimental evolution) and/or evolved (after experimental evolution) strain–plasmid pair. For each replicate within each combination, we estimated starting population densities based on plating (*Material and Methods* section *Measuring Plasmid Stability Traits* and *SI Appendix*, *Supplementary Model Information, section I*). We compared plasmid dynamics in the models to those observed in our experiment by comparing simulated and observed values of the time-averaged plasmid frequency (area under the curve, AUC, for the time series of plasmid frequency over time for 15 d). We implemented the model in R (v4.1.0), using the function “ode” in the package deSolve for numerical integration (72).

### Sequencing and Genomic Analyses.

Strains 1, 15, and 19 were previously sequenced with Illumina MiSeq (WGS, paired-end, 2 × 250 bp) and Oxford Nanopore MinION methods to generate hybrid assemblies (68). Ancestral and evolved clones of the strain–plasmid combinations generated here (Fig. 1) were Illumina sequenced (NextSeq500, paired-end, 2 × 150 bp). In brief, we grew single clones in monoculture with appropriate antibiotics overnight and extracted genomic DNA (QIAGEN Genomic DNA Kit) for library preparation with the Nextera XT Library Preparation Kit. We used fastp for quality control, adapter trimming, and quality filtering of reads acquired by Illumina sequencing (73). Because new strain–plasmid pairs could contain all plasmids from donor and recipient strains, we first generated the maximal reference sequence in silico against which we mapped new reads using breseq (v0.34.1) (74). This revealed that after generating the new strain–plasmid combinations, all nonfocal plasmids were retained in all strains. Further, we detected cotransfer of a nonfocal plasmid (alongside the ESBL plasmids) for a single nonfocal plasmid (ColRNAI from strain 1 when generating strain19-p1ESBL). Based on this, we generated the final reference sequences used to compare ancestral and evolved genomes. We mapped reads for mutation calling with the breseq pipeline (v0.34.1) and used gdtools to correct mutations by corresponding ancestral sequences. Predicted mutations and unassigned missing coverage/new junction evidence were curated manually for each sequenced clone using CLC Genomics Workbench 11 (Qiagen). To generate plasmid comparisons, we used the visualization tool Easyfig (v2.2.2) (75). We visualized the genome and sequencing data using Circos (v0.69) (76) and Geneious prime (v2022.1.1). For the ancestral ESBL plasmid-free strain 19, sequencing resulted in poor quality and we mapped evolved ESBL plasmid-free clones against the ancestral strain19-p1ESBL/-p15ESBL instead. Sequencing revealed contamination in one replicate population of Strain1-p15ESBL, which we excluded from further analysis.

### Statistics.

We performed statistical analyses using R (v4.1.0). To test strain and plasmid effects for plasmid success (AUC in the main experiment) and plasmid stability traits (transfer rates and growth costs), we performed analyses of variances by fitting linear models (function “lm”). To calculate AUC, for both simulated and observed plasmid frequencies over time, we used the function “sintegral” in the package Bolstad2 for numerical integration (77). In analyzing transfer rates, we assigned a dummy value of 10^−17^ (approximating the transfer rate if transconjugants would be present at the detection limit of ~200 CFU/mL) to the individual replicates where no transconjugants were detected (two replicates each for strain19-p1ESBL pink/green and the ancestral strain19-p1ESBL). Finally, to assess the fit of our different model variants, we performed linear regression of predicted and observed AUC. Note that for this, we excluded the replicate population 3/orange in Anc/Evo models, because of missing parameter estimates for this replicate, caused by the lack of Amp-susceptible clones at day 15.

## Supplementary Material

Appendix 01 (PDF)

Dataset S01 (XLSX)

Dataset S02 (XLSX)

Dataset S03 (XLSX)

Dataset S04 (XLSX)

Dataset S05 (XLSX)

Dataset S06 (XLSX)

Dataset S07 (XLSX)

## Data Availability

Datasets generated during this study are available from the data repository Dryad under the doi:10.5061/dryad.crjdfn38s (78). All study data are included in the article and/or *SI Appendix*.
