# Supplementary material for: Host-specific plasmid evolution explains the variable spread of clinical antibiotic-resistance plasmids
Source: Proc Natl Acad Sci U S A. 2023 Apr 6;120(15):e2212147120. doi: 10.1073/pnas.2212147120 (PMC10104558; doi:10.1073/pnas.2212147120)
Supplement: Supplementary file 1 — Appendix 01 (PDF) [file pnas.2212147120.sapp.pdf]

Supplementary Material

Supplementary material for: *Host-specific plasmid evolution explains the variable spread of clinical antibiotic-resistance plasmids*

Fabienne Benz[1,2,3*] and Alex R. Hall[1]

[1]Institute of Integrative Biology, Department of Environmental Systems Science, ETH Zurich, Zurich, Switzerland

Present address:
[2]Synthetic Biology Group, Department of Microbiology, Institut Pasteur, Paris, France
[3]Microbial Evolutionary Genomics Group, Department of Genomes and Genetics, Institut Pasteur, Paris, France

*Corresponding author
E-mail: fabienne.benz@env.ethz.ch; fabienne.benz@pasteur.fr

Contents:

1) Supplementary Methods .... p. 2
2) Supplementary Model Information .... p. 5
3) Supplementary Figures .... p. 9
4) Supplementary Tables .... p. 23
5) Supplementary Datasets .... p. 28
6) Supplementary References .... p. 35

1) Supplementary Methods

Verification of plasmid carriage in evolved clones

In addition to sequencing of evolved (endpoint, after 15d) clones (colony isolates), we verified the presence of ESBL-plasmids by PCR-testing three further Amp-resistant clones from each endpoint population (**Table S1**). Because plasmid regions selected for PCR verification could in theory change during the evolution experiment, we used up to two distinct sets of primers for each clone. We amplifed the *oriT* region of both IncIγ plasmids (pIncI_oriT_fw: AGTTCCTCATCGGTCATGTC/ pIncI_oriT_rv: GAAGCCATTGGCACTTTCTC) and used one primer pair specific to p1ESBL (p1ESBL_fw: GGAACCCGAAGTATCTCACC/ p1ESBL_rv: GGAGCACCAGAAAGTCCAC) or p15ESBL (p15ESBL_fw: AGGATGA-ACTGAAGGCAACG/ p15ESBL_rv: CCAGTAGATAAACCACATTGC). This allowed us to verify plasmid presence with at least one of the primer pairs for three clones of 22 of the 23 endpoint populations. For one population of strain 15-p15ESBL (purple), we could not confirm plasmid carriage in the three tested clones with these primers, although the sequenced clone from this population did carry the plasmid.

Verification of strain identity in endpoint populations

We verified strain identity for multiple colonies from each endpoint population. Because strains 1, 15 and 19 belong to different sequencing types and phylogroups we could differentiate them by Clermont phylo-typing (**Table S2**) (1, 2). For strain 1, this resulted in amplification of *chuA* (288bp, indicating group F), for strain 15 in amplification of *ArpA* (400bp, indicating B1), and for strain 19 *chuA* and *yjaA* (211bp, indicating group B2). For each endpoint population, we tested three colonies, three per colony morphology in case of multiple distinct colony morphologies, and repeated the evolution experiment for replicate populations where contamination was suspected. For the strain1-p15ESBL_green population, we noticed the contamination only later and therefore this population was excluded entirely. We verified that plasmid-free control populations remained susceptible to Amp throughout the whole experiment by selective plating of undiluted cultures. In cases where we found growth, we concluded cross-contamination with plasmid-containing populations and repeated the evolution experiment for those replicates.

Construction of new strain-plasmid combinations

Generated strain-plasmid pairs are shown in **Fig. 1** (also see **Table S3**). To cure strains 1, 15 and 19 of their IncIγ-plasmids, we used replicon incompatibility and challenged each strain with the temperature-sensitive and Cm resistant pCP20-IncI1, encoding the replicon of p15ESL (3). To cure p19ESBL, we used the Cm resistant pCure2 (4) competing with IncF replicons. We electroporated curing plasmids into the strains to be cured, plated them on the respective antibiotic for their overnight selection, and verified ESBL-plasmid loss by restreaking on selective plates and colony PCR in case of strain19-IncIγ. Curing plasmids were lost by growing ESBL-plasmid-cured colonies overnight at 37°C without antibiotic selection. We then combinatorially re-introduced focal ESBL plasmids into each *E. coli* host strain, by first conjugating strains 1 and 15 with the *Salmonella enterica* Typhimurium (S.Tm) strain ATCC 14028 *marT::cat* (5) to generate an intermediate host. Transferring p1ESBL and p15ESBL from S.Tm to the ESBL-plasmid cured recipients allowed the distinction of donor and recipient by plating on MacConkey agar (lac negative S.Tm forming yellow colonies and lac positive *E. coli* forming red colonies).

Construction of plasmid mutants and expression vector

To test the effect of mutations in the *ssi3* operon of the plasmid leading regions, as we have observed them in both ESBL-plasmids with strain 15, we generated the two knockout mutants, mutA and mutB (**Fig. 7A** and **B**). For mutA we deleted the 2049bp large gene *yfhA*, thereby truncating the overlapping C-terminal of the hypothetical protein encoded by *ykfF* (34 nt). For mutB we deleted *yfhA* and *psiA*/*psiB*, thereby truncating the genes *ykfF* and the N-terminal coding region of *ygaA*, encoding a hypothetical protein (removed 3 nt but retained a start codon). To generate these mutants we used the lambda red recombinase system with pKD4 as a template for the Kan resistance marker (6) (primers in **Table S5**). For the mutant construction, we conjugated p1ESBL and p15ESBL to *E. coli* DH10B with pSIM18 (pSIM18 encodes λ Red recombination genes, facilitating homologous recombination, resistant to Hygromycin (7)). We PCR verified Amp/Kan resistant colonies for insertion of the resistance marker. To remove the Kan cassette we introduced pFLP2, which encodes a FLP-recombinase and for which we replaced the Amp resistance marker with Cm (8). For this, we grew strains containing the mutant plasmids at 37°C, leading to loss of the temperature-sensitive pSIM18 and allowing use of pSIM18 as a marker plasmid in strains 1/15/19ΔpESBL, for introduction of p1ESBLmutA/B and p15ESBLmutA/B via conjugation. We were not able to conjugate p1ESBLmutA/B and p15ESBLmutA/B into strain 19, which was also the case for the control-transferred WT

p1ESBL/ p15ESBL in this experimental block. Therefore, we performed subsequent conjugation assays only with strains 1 and 15 (**Fig. 7C**). For the complementation assay, we cloned *yfhA* from p1ESBL on the expression vector pHerd30T (9), downstream of promoter pBAD employing USER cloning (New England Biolabs,(10)). In brief, we amplified the *yfhA* and pHerd30T by PCR with the Phusion U Hot Start DNA Polymerase (Thermo), employing primers with overhangs containing a Uracil base (primers p1ESBL; pFB135: acatacccAUGCCATCCGTGATTTCGGG/ pFB137: acggccagUtcaggcg-gcatcagccag, primers pHerd30T; pFB136: atgggtatgUatatctccttcttaaag/ pFB6 actggccgucgttttac, (5’>3’)).

2) Supplementary Model Information

We used a mathematical model with two main variations: the ancestral (Anc) model, considering either ancestral or evolved plasmid-stability traits as model parameters, and the evolved (Evo) model.

I) The ancestral model

To simulate plasmid dynamics during 15-days of serial passage, assuming plasmid-carrying and plasmid-free subpopulations maintain their ancestral phenotypes (the phenotypes of the different ancestral strain-plasmid combinations and plasmid-free equivalents prior to the 15-day experiment), we used existing models (11, 12). Because the donor and recipient strains in each of our replicate populations were otherwise identical (to the best of our knowledge), we assumed transconjugants to be identical to donors, which allowed us to simplify the original notation with three subtypes (donors, recipients and transconjugants) to two subtypes (plasmid carrying, N_P, and plasmid-free, $N_\emptyset$, where N denotes the density of given cell type; **Fig. S4**). Note this change in the notation simplifies the equations and plotting, but does not affect the results (if we instead use the original models but sum the abundances of donors and transconjugants together, we obtain the same output). In these models, each subtype replicates at their specific growth rate ψ, constrained by nutrient-limitation (Monod function), and plasmids can transfer at rate γ to plasmid-free cells. We estimated both parameters for each strain-plasmid pair experimentally (**Fig. 3A-B**), and starting densities of N_P and $N_\emptyset$ ($t = 0$) for each replicate population by plating. To simulate serial passage, each 15-day simulation included 15 sequential growth cycles, with each growth cycle except the first one initiated with 0.001 times the abundances from the preceding cycle. We also made a version of the Ancestral Model using parameters from evolved strains (that is, parameters measured for evolved p^+ and p^- clones from each replicate population, rather than the ancestral strains), resulting in the updated Anc Model Evo Parms model (**Fig. 5B)**.

II) The evolved model

We made an additional version of the model (Evo Model; eq. 4-10; **Fig. S5**) that assumes mutant (evolved) strains/plasmids to be present at initially very low frequencies, thereby simulating the spread of mutant strains/plasmids from rare more realistically than Anc Model Evo Parms above. The Evo model simulates population density, *N*, for six possible cell types: *AØ*, *EØ, AA*, *AE*, *EA*, *EE*, with the first letter denoting the bacterial host (*A*=Ancestral,

E=Evolved) and the second letter the plasmid (*Ø*=none, *A*=Ancestral, *E*=Evolved). We included mutant strains with mutations on the plasmid (*AE*), chromosome (*EØ* or *EA*) or both (*EE*), at an initial abundance of one cell at the start of the experiment (at $t = 0$). We empirically estimated ψ and γ for ancestral strains without and with ancestral plasmids (*AØ*, *AA*) and evolved strains without and with evolved plasmids (*EØ*, *EE*). We could only measure the plasmid stability traits ψ and γ for either fully ancestral or fully evolved pairs. We therefore made two versions of the Evo Model, each making different assumptions for the growth rates of *EA* and *AE* bacteria. (i) In Evo Model 1 (**Fig. 5C)**, the evolutionary state of the plasmid decides the growth rate. Therefore $\psi_{EA} = \frac{\psi_{AA}}{\psi_{A\emptyset}}\psi_{E\emptyset}$ and $\psi_{AE} = \frac{\psi_{EE}}{\psi_{E\emptyset}}\psi_{A\emptyset}$. That is, *EA* grows like *EØ*, but pays the same cost of carrying the ancestral plasmid as *AA* does; likewise, *AE* grows like *AØ*, but pays the same cost of carrying the evolved plasmid as *EE* does. (ii) In Evo Model 2, the evolutionary state of the bacterium decides the growth cost of plasmid carriage for combinations not measured directly. Therefore, in this version of the model, $\psi_{EA} = \psi_{EE}$ and $\psi_{AE} = \psi_{AA}$. In both versions, Evo Models 1 and 2, we assumed transfer rate γ for the subtypes *EA* and *AE* to be determined by the evolutionary state of the plasmid and therefore $\gamma_{\mathrm{EA}} = \gamma_{AA} = \gamma_A$ and $\gamma_{AE} = \gamma_{\mathrm{EE}} = \gamma_E$. We found that Evo Model 2 supported the same conclusions as Evo Model 1 (simulated vs observed plasmid success: $R^2 = 0.25$, $p < 0.05$; **Fig. S7**).

$$\frac{\mathrm{d}N_{A\emptyset}}{dt} = \psi_{A\emptyset}\frac{C}{q+C}N_{A\emptyset} - \gamma_A\frac{C}{q+C}N_{A\emptyset}(N_{AA}+N_{EA}) - \gamma_E\frac{C}{q+C}N_{A\emptyset}(N_{AE}+N_{EE}) \quad (4)$$

$$\frac{\mathrm{d}N_{AA}}{dt} = \psi_{AA}\frac{C}{q+C}N_{AA} + \gamma_A\frac{C}{q+C}N_{A\emptyset}(N_{AA}+N_{EA}) \quad (5)$$

$$\frac{\mathrm{d}N_{AE}}{dt} = \psi_{AE}\frac{C}{q+C}N_{AE} + \gamma_E\frac{C}{q+C}N_{A\emptyset}(N_{AE}+N_{EE}) \quad (6)$$

$$\frac{\mathrm{d}N_{E\emptyset}}{dt} = \psi_{E\emptyset}\frac{C}{q+C}N_{E\emptyset} - \gamma_A\frac{C}{q+C}N_{E\emptyset}(N_{EA}+N_{AA}) - \gamma_E\frac{C}{q+C}N_{E\emptyset}(N_{EE}+N_{AE}) \quad (7)$$

$$\frac{\mathrm{d}N_{EA}}{dt} = \psi_{EA}\frac{C}{q+C}N_{EA} + \gamma_A\frac{C}{q+C}N_{E\emptyset}(N_{EA}+N_{AA}) \quad (8)$$

$$\frac{\mathrm{d}N_{EE}}{dt} = \psi_{EE}\frac{C}{q+C}N_{EE} + \gamma_E\frac{C}{q+C}N_{E\emptyset}(N_{EE}+N_{AE}) \quad (9)$$

$$\frac{\mathrm{d}C}{dt} = -(\psi_{A\emptyset}N_{A\emptyset} + \psi_{AA}N_{AA} + \psi_{AE}N_{AE} + \psi_{E\emptyset}N_{E\emptyset} + \psi_{EA}N_{EA} + \psi_{EE}N_{EE})e\frac{C}{q+C} \quad (10)$$

III) Resource availability and the resulting population densities

For all the model variations above, we set the amount of resources required per cell division to $e = 1$, which allows for maximal population densities identical to the initial resource concentration C_0. For the half-velocity constant, we assumed $q = 0.5 \times C_0$. For Anc and Evo

models, we set $C_0 = 1.15 \times 10^{12}$ and therefore the number of cells produced per growth cycle $N = 1.15 \times 10^{12}$. This setting predicted plasmid dynamics close to the observed plasmid frequencies, for example with some plasmids spreading and others declining in frequency, while overestimating total population densities. An alternative model, where we estimated C_0 by calculating the average of total population density enumerated on days 1,3,5,10, and 15 ($N = 1.15 \times 10^9 = C_0$, **Fig. S6**), yielded reasonable total population sizes, but consistently underestimated plasmid frequencies. For example, in this version of the Anc Model all plasmids were eventually lost (albeit at variable rates), unlike in our 15-day experiment. Despite this, the predictions arising from these two versions of the model (with larger and smaller total population sizes, due to different values of C_0) were in general similar (correlation of simulated AUCs: $r = 0.7$, $p < 0.001$ for Anc Model; $r = 0.52$, $p < 0.05$ for Anc Model Evo Parms; $r = 0.42$, $p = 0.05$ for Evo Model 1). For example, both versions of the Evo Model 1 captured spread of strain 19-p1ESBL better than both ancestral versions. Note these models, as in previous work with the same base model (11), assume no transfer in stationary phase. This assumption potentially contributes to the lower-than-observed average plasmid frequencies simulated with realistic total population sizes (because if there is in fact some stationary phase transfer, the resulting transconjugants are not accounted for by such a model). We describe an alternative version of the model below that relaxes this assumption, allowing transfer in stationary phase.

For some individual replicates, such as some with strain 19-p15ESBL, Anc and Evo models both performed poorly. In these cases growth rates of evolved p^+ clones relative to corresponding evolved p^- clones were low compared to in other replicates (**Fig. 4A**). Underprediction in such cases could potentially arise due to effects of limited sampling (isolation of single evolved p^+ and p^- colonies), but we do not rule out other factors not accounted for in these types of models, such as variable plasmid loss rates (13). Any such effects did not prevent the Anc Model Evo Parms and Evo Models from making relatively good predictions overall.

IV) Relaxing stationary growth phase assumptions for γ

As in earlier work using the same models, the Monod function ($\frac{C}{q+C}$) in the transfer term (terms including γ) means there is no plasmid transfer when there are no resources left (i.e., assumes no stationary-phase transfer). For IncF-family plasmids, transfer is known to be reduced when bacterial growth is inhibited (14, 15), suggesting this assumption is at least sometimes

reasonable. How this translates to other plasmid types and growth conditions is not well understood. Therefore, the transfer assumption may be overly stringent in some cases, and a possible explanation for underestimation of average plasmid frequency in our models above that otherwise reproduced total population growth relatively realistically. To test this, we detached plasmid transfer from resource availability in the Anc Model and Evo Model 1. We did this by removing ($\frac{C}{q+C}$) from the transfer terms in each equation (Anc Model; eq. 1-2, Evo Model; eq. 4-9). With realistic total population sizes ($C_0 = 1.15 \times 10^9 = N$), the predictions of this version of the model were identical to the stricter versions above which assumed no stationary phase transfer (correlation between simulated AUC values for the two model versions: r = 1 for both the Anc model and Evo Model 1). With $C_0 = 1.15 \times 10^{12}$ (resulting in more total population growth), the model allowing stationary phase transfer differed from the more stringent version and performed relatively well (**Fig. S8**). Despite the good performance of this model, it should be interpreted with caution, because it assumes both unrealistic total population growth and unrestrained stationary phase transfer. Nevertheless, this suggests future work with variations of these widely used population dynamic models should consider the possibility that this assumption may result in underestimation of plasmid transfer across the whole growth cycle.

3) Supplementary Figures

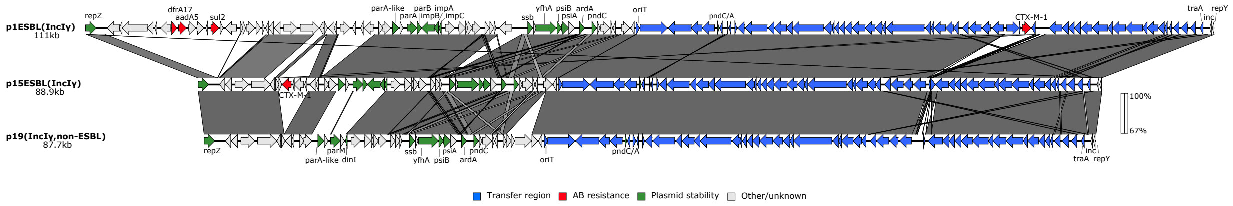

Figure S1: Comparison of IncIγ plasmids natively associated with the three clinical strains used in this study. All three strains (1, 15, 19) were natively associated with IncIγ plasmids (sequences shown here). The IncIγ plasmids from strains 1 and 15 (p1ESBL and p15ESBL; the top two sequences in this plot) encode ESBL phenotypes and were included in our experiments. The IncIγ plasmid from strain 19 (the bottom sequence in this plot) does not encode an ESBL-gene and was therefore not included in our experiments, but is shown here to provide background information about the plasmids native to each strain. The three plasmids show high sequence similarity in their backbone. Gene functions are given in color (see legend at bottom), and for simplicity only selected genes are labelled.

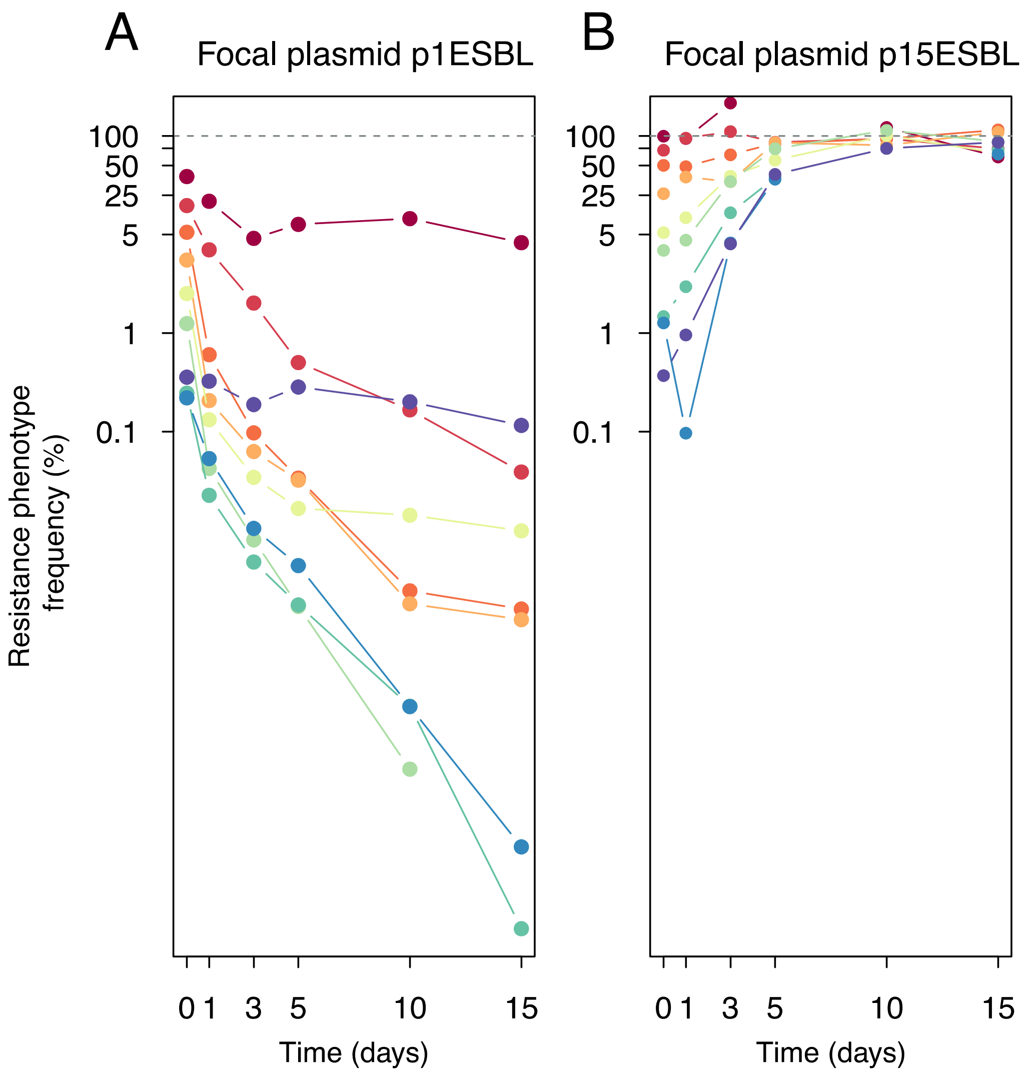

Figure S2: Testing whether initial plasmid frequency affects plasmid dynamics for strain 19 with p1ESBL (A) and p15ESBL (B) during an additional 15-day experiment. The key result here is that across a range of starting frequencies, p1ESBL declined during this 15 day experiment and p15ESBL increased in frequency. This shows any average difference in starting frequency in the main experiment is unlikely to have explained the difference in plasmid success in **Fig. 2**. We performed this additional evolution experiment in the same way as that described in the main text (**Fig. 2**; Methods), with a slightly different plating scheme to estimate the fraction of resistant phenotypes (separate plating on LB and Amp agar, rather than replicate-plating). For Strain19-p1ESBL, we detected significant plasmid loss already after overnight culture in the presence of Amp prior to inoculation. This is in line with our other, short-term plasmid stability assays (**Fig. S3/S9**). For both strain-plasmid pairs (A&B), we varied the starting plasmid fraction by varying the ratios of pure cultures made from plasmid-carrying and plasmid-free strains mixed together at the start.

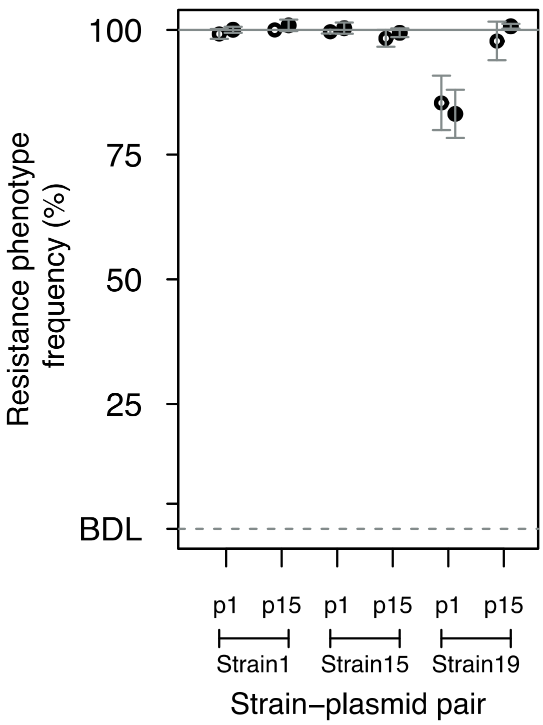

Figure S3: Stability of plasmids in each ancestral strain-plasmid combination over 24 hours. The frequency of plasmid-carrying cells was estimated by selective plating. For each strain-plasmid combination, empty circles show the fraction of plasmid-carrying cells at t = 0 h and full circles at t = 24 h. Each culture was inoculated from a pure-culture of a plasmid-carrying strain, so any net loss reflects significant segregational loss (relative to possible re-acquisition via conjugation in the same cultures; see Methods). The relatively low initial frequency for strain19-p1ESBL is consistent with our other observations (**Fig. S2/ S9**), suggesting possible plasmid loss during cultivation prior to inoculation. Despite this, this plasmid was stable after inoculation into the experimental conditions here (similar frequency at t = 0 h and at t = 24 h). Stability (the change in frequency over 24 h) was not dependent on strain or plasmid (two-way ANOVA with frequency at t = 24 h relative to frequency at t = 0 h as the response variable, effect of plasmid: $F_{1,25} = 0.355$, $p > 0.05$; effect of strain: $F_{2,25} = 0.011$, $p > 0.05$).

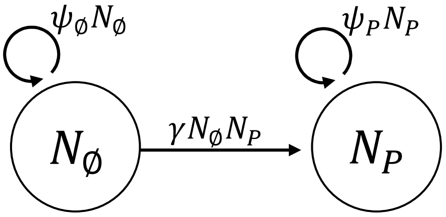

Figure S4: Schematic diagram of the Ancestral (Anc) model for plasmid population dynamics (eq. 1-3). $N_{\emptyset}$ denotes plasmid-free cells and N_P plasmid-carrying cells. Arrows indicate transition of cells between states, where ψ is bacterial replication rate and γ is the horizontal plasmid transfer rate. Note the Monod function ($\frac{C}{q+C}$) was omitted here from arrow labels.

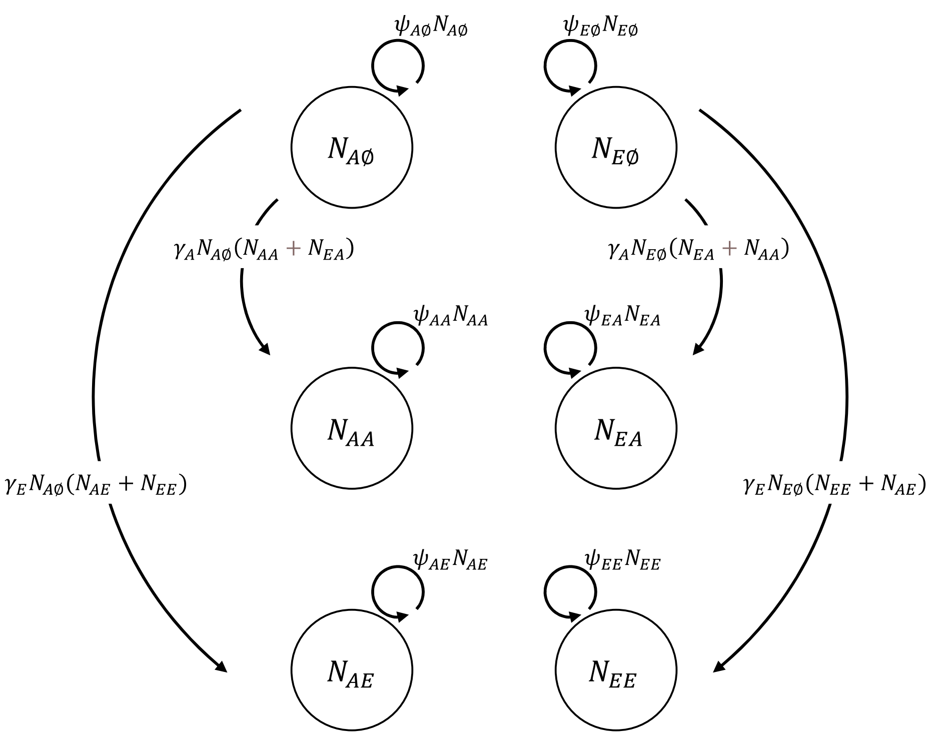

Figure S5: Schematic diagram of the Evolved (Evo) Model for plasmid population dynamics, which includes ancestral and evolved strain-plasmid pairs (eq. 4-10). *N* gives the population density of a given cell type, with the first subscript indicating the bacterial state (*A* for ancestral, *E* for evolved) and the second subscript the plasmid state (*A* for ancestral, *E* for evolved, and *∅* for absent) for each type. Arrows indicate transition of cells between states, as above in **Fig. S4** and as described in the Supplementary Model Information section. Note the Monod function ($\frac{C}{q+C}$) was omitted here from arrow labels.

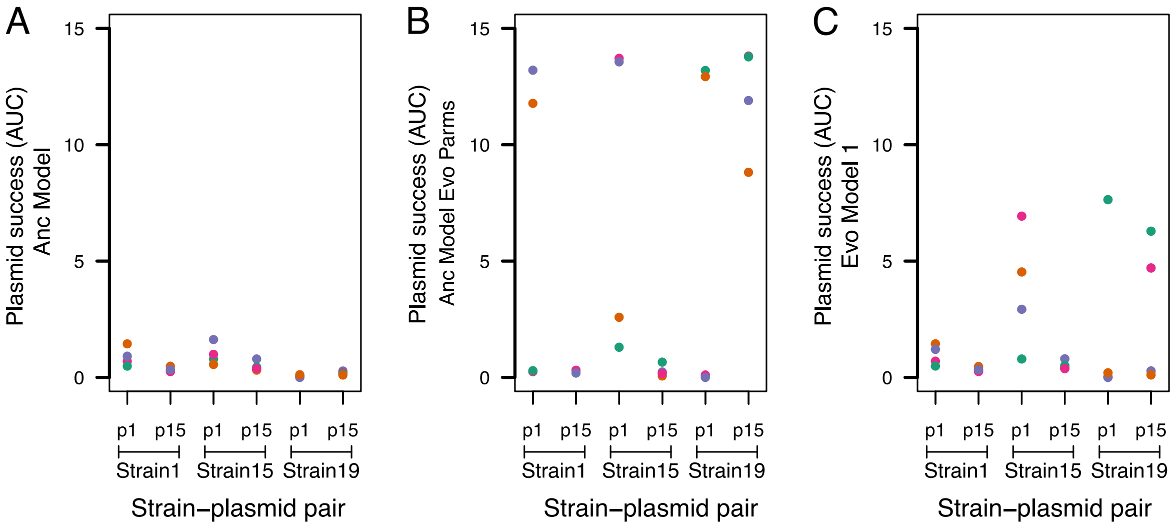

Figure S6: Plasmid success over time (area under the curve, AUC, from plasmid frequency over 15 days) for each replicate population in each strain-plasmid combination (x-axis), from simulations with alternative versions of each model. Here the total population size is smaller than in the versions shown in the main text (because here we set $C_0 = 1.15 \times 10^9$). (**A**) Anc Model, (**B**) Anc Model Evo Parms, (**C**) Evo Model 1. Colors show replicate populations.

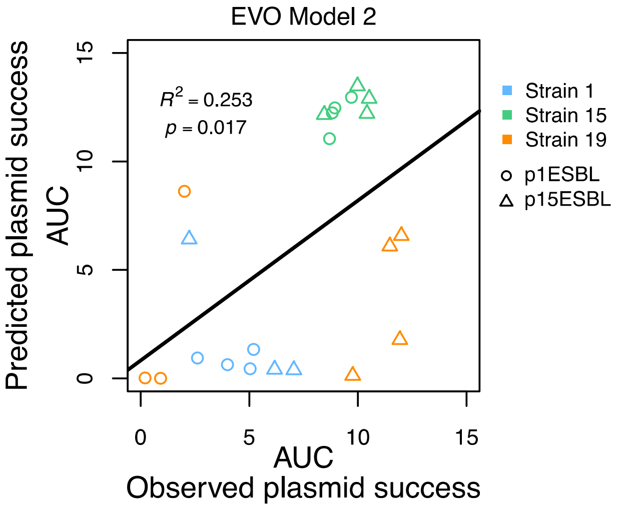

Figure S7: Observed plasmid success over 15 days vs predictions from the Evo Model 2. Plasmid success (area under the curve, AUC, from plasmid frequency over time) observed in our 15-day serial passage experiment (x-axis) is plotted against values predicted by the Evo Model 2, where the evolutionary state of the bacterium decides the growth cost of plasmid carriage for combinations not measured directly (growth rates and transfer rates measured for evolved clones isolated at the end of the experiment; y-axis).

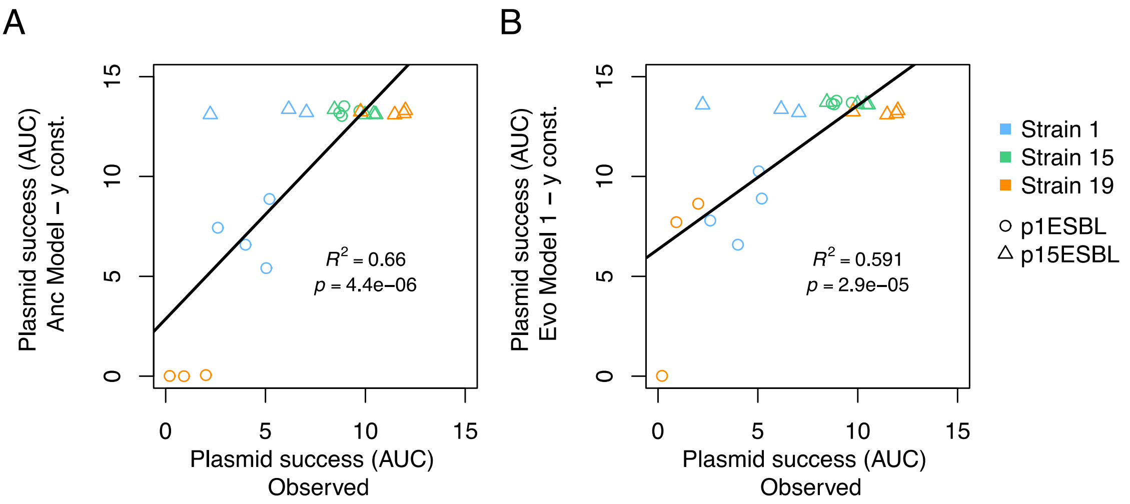

Figure S8: Observed plasmid success over 15 days vs predictions from (**A**) the Anc Model and (**B**) the Evo Model 1, but allowing for plasmid transfer in stationary phase. Plasmid success (area under the curve, AUC, from plasmid frequency over time) observed in our 15-day serial passage experiment (x-axis) is plotted against values predicted by alternative versions of the Anc and Evo Models (A&B), where the assumption that plasmid transfer is coupled to bacterial growth is relaxed (see Supplementary Model Information IV). Note these models assume both unrealistic total population growth ($C_0 = 1.15 \times 10^{12}$) and unrestrained plasmid transfer, and should therefore be interpreted very cautiously. These assumptions explain the higher average plasmid frequencies (higher AUC) for successful combinations here compared to other versions. This in turn helps to explain the similar performance of Anc and Evo versions here (successful combinations already spread very rapidly in the Anc Model (top-right in panel A), so evolutionary changes increasing plasmid transfer rates or reducing growth costs that are incorporated in the Evo Model have little impact on predicted dynamics).

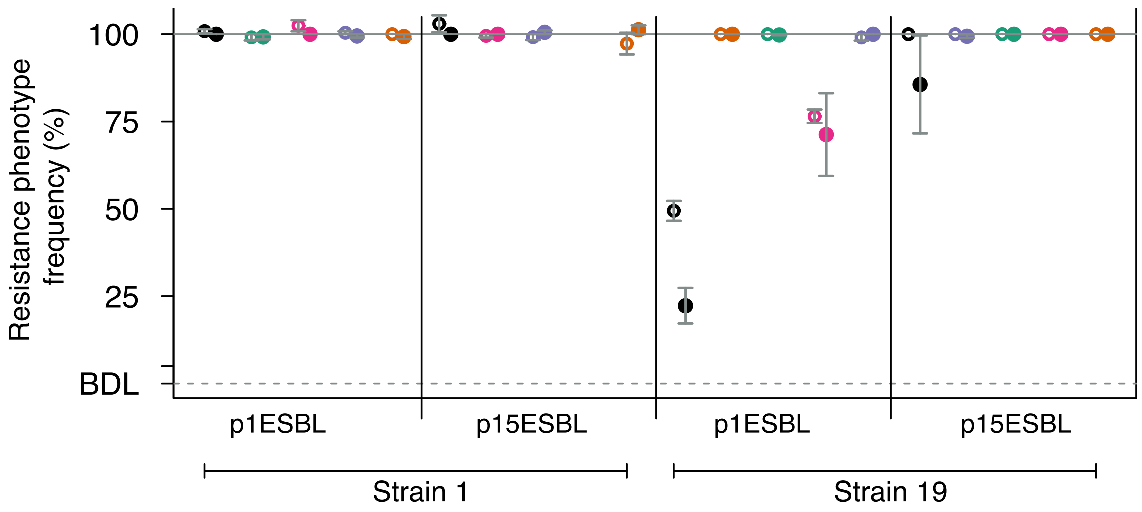

Figure S9: Plasmid stability over 24 hours for ancestral and evolved clones. The frequency of resistant colonies (approximating plasmid frequency) was estimated by plating at the start and end of 24 h incubation without antibiotics for strains 1 (left half) and 19 (right half) with p1ESBL and p15ESBL. Black points indicate the ancestral clone in each combination; other colours indicate evolved clones, with different colours indicating which replicate population each evolved clone was isolated from (as in other figures). For each clone (each pair of adjacent points), empty and full circles show the fraction of plasmid-carrying cells at $t = 0$ h and $t = 24$ h, respectively. The ancestral clone of strain 19 (black) was the only clone showing clear loss of plasmids during the assay (from ~50% to ~20% for p1ESBL, and from ~100% to ~80% for p15ESBL). This is consistent with relatively low frequencies upon inoculation observed in other experiments (**Fig. S2 & S3**). However, among evolved clones all plasmids were highly stable. There was no evidence that stability (taken as frequency at 24 h relative to at 0 h for each replicate assay) varied among the different evolved clones within each strain-plasmid combination (one-way ANOVA within each of the four strain-plasmid combinations: $p > 0.05$ in all cases), or on average among the different combinations (two-way ANOVA with stability as the response variable, taking the average for each evolved clone: effect of plasmid: $F_{1,12} = 3.414$, $p > 0.05$; effect of strain: $F_{1,12} = 1.405$, $p > 0.05$).

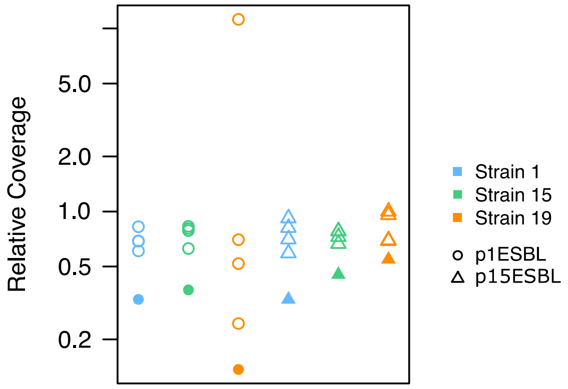

Figure S10: Coverage for ESBL-plasmid sequences relative to corresponding chromosomal sequences. Relative coverage (y-axis) is calculated as mean ESBL-plasmid coverage relative to mean chromosomal coverage for each clone in each strain-plasmid combination (see legend), with mean read-depth coverage estimated by breseq (16). Evolved clones (empty symbols) consistently had higher ESBL-plasmid relative coverage compared to ancestral clones (6 out of 6 cases; filled symbols). In all cases except one, the difference was moderate. These moderate differences should be interpreted with caution, because sequence preparation and coverage estimation may be subject to different biases for plasmids and chromosomes. However one evolved clone of strain 19-plasmid 1 showed a much larger difference in relative coverage compared to the ancestor. For this evolved clone, this suggests an increased copy number of p1ESBL, which coincided with an increase in transfer rate of several orders of magnitude (**Fig. 4B**, evolved replicate 3/purple). These findings are also in line with a recent study demonstrating increased plasmid transfer rates after evolutionary increases in copy number (17).

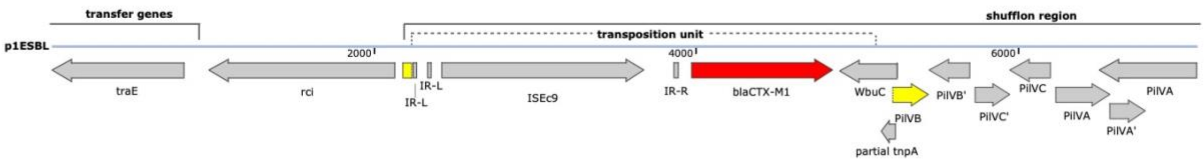

Figure S11: Shufflon region of the ancestral p1ESBL (position 88’151-96’017). A transposition unit containing an ISEc9 and $bla_{\text{CTX-M1}}$ (red) disrupts pilVB (yellow) within the shufflon segment B. In one evolved clone from the end of our 15-day serial passage experiment (**Fig. 2**; strain 19-p1ESBL replicate 2), this transposition unit had jumped into the chromosome (in *astA*, aerobic arginine catabolism; **Fig 6C,** orange), and the plasmid itself had been lost. In two other evolved clones (strain 19-p1ESBL replicate 4, pink in **Fig. 6C** and strain 1-p1ESBL replicate 3, purple in **Fig. 6A**), the entire transposition unit was inverted.

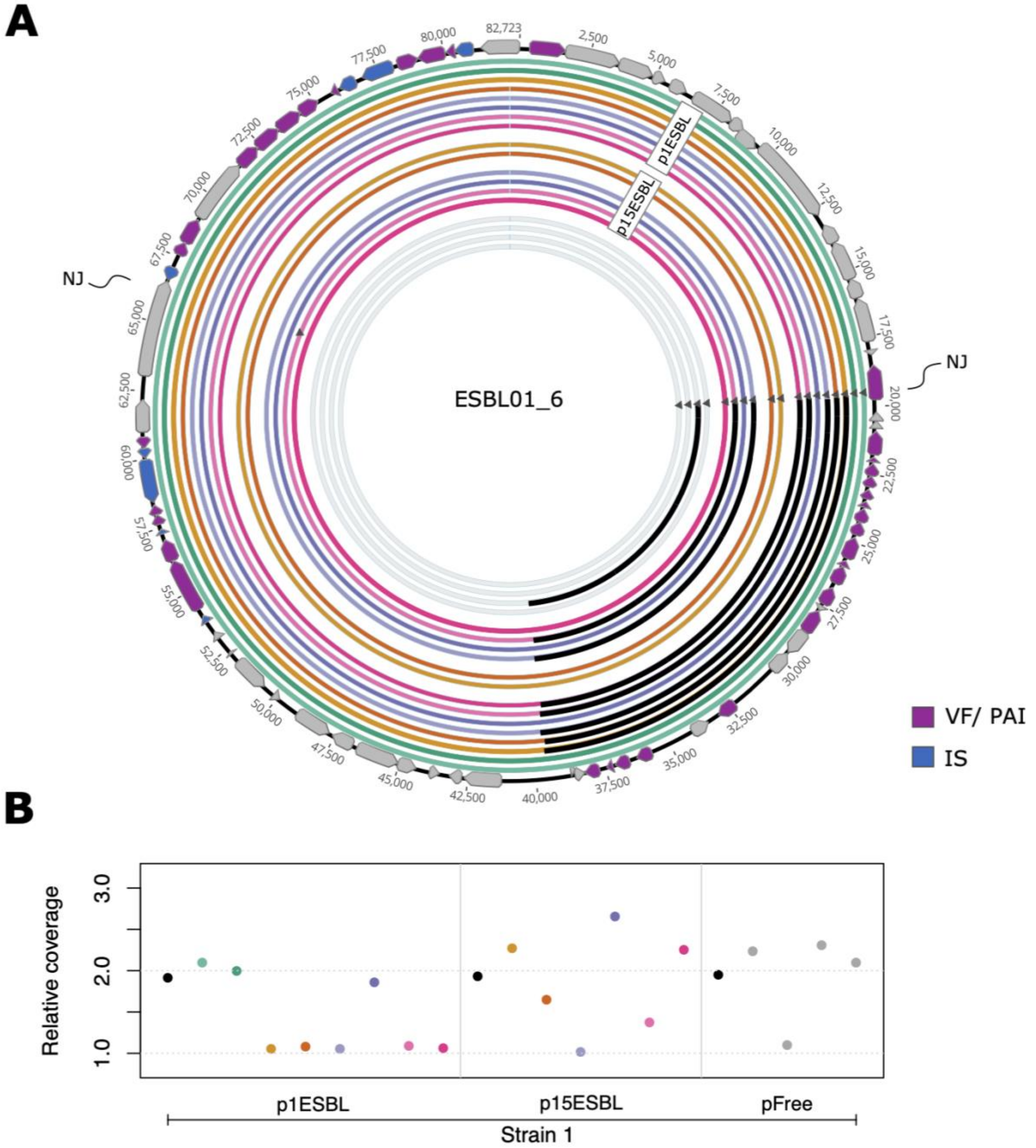

Figure S12: Legend on the following page

Figure S12: Deletions in a putative circularized pathogenicity island (PAI) in strain 1. **A)** With long-read sequencing we have previously determined the contig ESBL01_6 to be circularized in the original clinical isolate (18). Resequencing (Illumina) of the ancestral and evolved clones used in this study revealed a NJ (triangle) with the end of ESBL01_2, one of the two chromosomal contigs. This indicates that ESBL01_6 can exist both as part of the chromosome, potentially closing the gap between the two chromosomal contigs ESBL01_1 and ESBL01_2, and in circularized form. Black bars indicate ~20kb deletions and the colour of annotated genes indicates their classification determined by VrProfile (purple = virulence factor and/ or pathogenicity island, blue = insertion sequence, **Dataset S5**) (19). **B)** Sequence coverage (Illumina) of ESBL01_6 relative to the average of the other chromosomal contigs indicates ESBL01_6 to be present either as a single or two copies. This coincides with deletions in panel A: two copies are present in the absence of the deletion and only one copy is present in case of a deletion in the PAI.

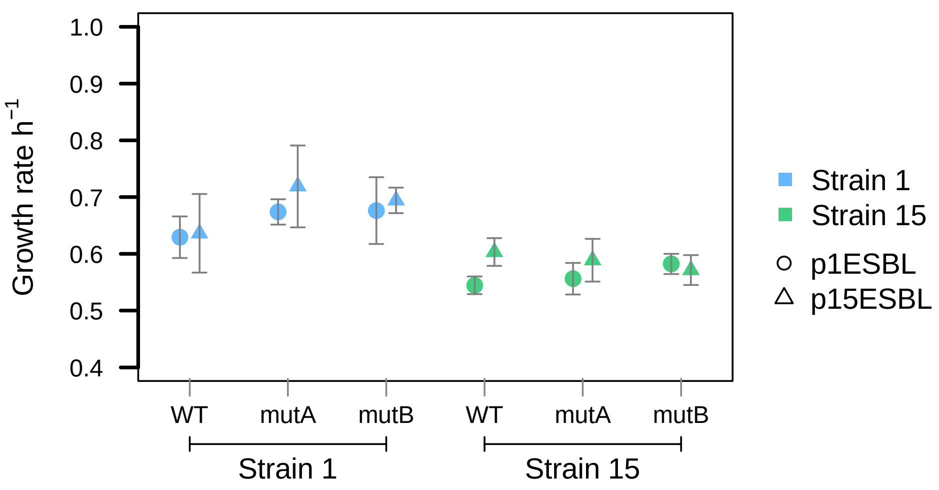

Figure S13: Generated leading-region mutations (mutA, mutB) have no significant effect on bacterial growth rate relative to the wild type (WT), measured in the absence of antibiotics over 24h (Welch Two Sample *t*-test, $p < 0.05$ in all cases before and after Holm's correction for multiple testing).

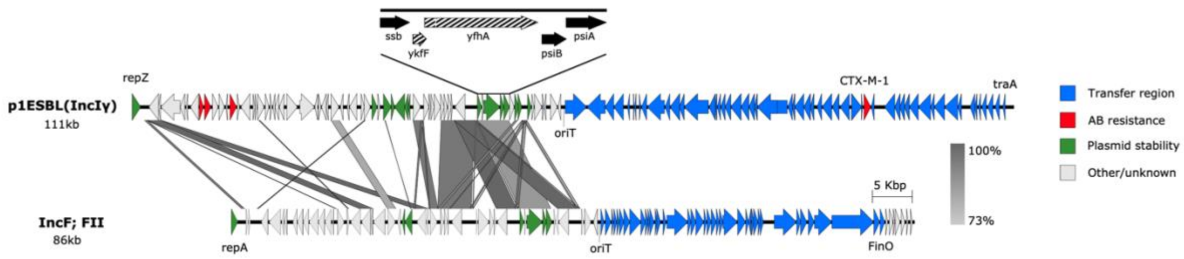

Figure S14: High sequence similarity of the *ssi3* operon on p1ESBL (shown here; top) and p15ESBL (full sequence shown in Fig. S1) with the *ssi3* operon on the IncF plasmid native to strain 1 (bottom). The *ssi3* operon has been shown to be largely conserved among conjugative plasmids in *E. coli* (20).

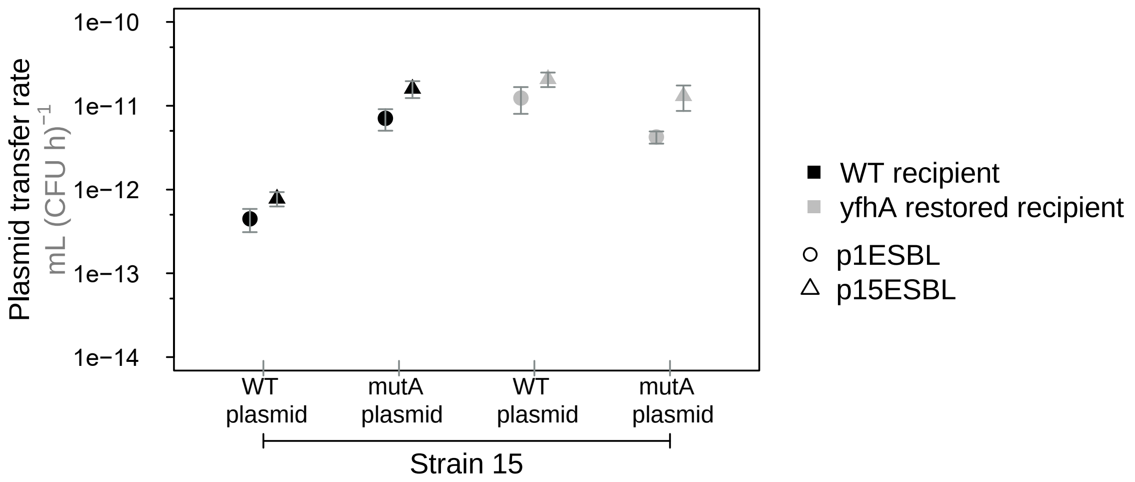

Figure S15: Effects of complementing *yfhA* in recipient strains on plasmid transfer-rates of wild-type and mutA plasmids. We conjugated wild-type (WT) and mutA (**Fig. 7B**) versions of plasmids p1ESBL and p15ESBL from strain 15 donors to marked strain 15 recipients, with or without complementation of *yfhA* in the recipient strains (see Methods). Complementation increased transfer rates for WT plasmids, but did not affect transfer of mutant plasmids (two-way ANOVA, complementation × strain (WT/mutA) interaction: $F_{1,\ 28} = 18.434$, $p < 0.001$; main effects NS). This suggests a positive effect of excess YfhA on transfer, but only if the WT *ssi3* operon is present.

4) Supplementary Tables

Table S1: Verification of ESBL-plasmids presence in three independent Amp resistant clones from each endpoint population. Primers used to verify plasmid presence/ absence. Yes/ no indicates successful/ unsuccessful amplification of indicated regions.

Strain-plasmid pair	Population ID	Population colour	oriT amplicon	plasmid specific amplicon
s1_p1	22	green	yes	not tested
s1_p1	50	orange	yes	not tested
s1_p1	53	purple	yes	not tested
s1_p1	55	pink	yes	not tested
s1_p15	2	orange	yes	not tested
s1_p15	7	pink	yes	not tested
s1_p15	29	green	yes	not tested
s1_p15	40	purple	yes	not tested
s15_p1	6	orange	not tested	yes
s15_p1	39	green	yes	yes
s15_p1	51	purple	not tested	yes
s15_p1	43	pink	not tested	yes
s15_p15	24	orange	yes	no
s15_p15	27	pink	yes	no
s15_p15	37	green	yes	no
s15_p15	46	purple	no	no
s19_p1	4	purple	not tested	yes
s19_p1	17	pink	not tested	yes
s19_p1	31	green	not tested	yes
s19_p1	41	orange	yes	no
s19_p15	1	orange	not tested	yes
s19_p15	13	green	not tested	yes
s19_p15	18	purple	not tested	yes
s19_p15	26	pink	not tested	yes

Table S2: Primers used for quadruplex PCRs for Clermont phylo-typing to verify strains 1, 15, and 19 (see Supplementary Methods).

Primer	Direction	Target	Sequence (5’> 3’)
chuA.1b	fw	*chuA*	ATGGTACCGGACGAACCAAC
chuA.2	rev	*chuA*	TGCCGCCAGTACCAAAGACA
yjaA.1b	fw	*yjaA*	CAAACGTGAAGTGTCAGGAG
yjaA.2b	rev	*yjaA*	AATGCGTTCCTCAACCTGTG
TspE4C2.1b	fw	TspE4.C2	CACTATTCGTAAGGTCATCC
TspE4C2.2b	rev	TspE4.C2	AGTTTATCGCTGCGGGTCGC
AceK.f	fw	*arpA*	AACGCTATTCGCCAGCTTGC
ArpA1.r	rev	*arpA*	TCTCCCCATACCGTACGCTA

Table S3: Clinical *E. coli* strains (sequence type, ST, in brackets) and plasmids used to construct the strain-plasmid combinations used in this study. For each strain, each row gives one plasmid, identified by its replicon type(s). ESBL plasmids are indicated by strain-specific names in brackets; p1ESBL and p15ESBL are conjugative, p19ESBL is not and was cured from strain 19 to allow selection of p1ESBL and p15ESBL. To facilitate the introduction of p1ESBL and p15ESB into strain 19, IncIγ was also cured from strain 19. Resistance determinants: p1ESBL encodes for *aadA5*, $bla_{\text{CTX-M-1}}$, *dfrA17* and *sul2*, p15ESBL for $bla_{\text{CTX-M-1}}$ and p19ESBL for $bla_{\text{CTX-M-27}}$, *tet(A)*, *aph(6)-Id*, *aph(3'')-Ib*, *sul2*, *mph(A)*, *sul1*, *aadA5*, d*frA17*, *ant(3'')-Ia*. With these three strains and p1ESBL and p15ESBL we created a fully factorial set of six strain-plasmid combinations. The star (*) with the IncFIC(FII) replicon in strain 1 indicates predictive uncertainty because the blast hit to IncFIC(FII) was shorter than < 75% coverage (18).

Strain 1 (ST 117)		Strain 15 (ST 40)		Strain 19 (ST 131)	
**Replicon**	**Size (kb)**	**Replicon**	**Size (kb)**	**Replicon**	**Size (kb)**
IncIγ (p1ESBL)	111	IncIγ (p15ESBL)	88.9	IncIγ (cured)	87.7
IncFIA;IncFIB; IncFIC(FII)	153.9	Col(MGD2); ColRNAI	5.3	IncFII;IncFIB; IncFIA;Col156 (p19ESBL)	130
IncFIC(FII)*	86	Col156	4.7	Col156	5.2
ColRNAI	7			Col(MG828)	1.5

Table S4: Phage prediction for strain 19, performed with Phaster (22). Note that only the first of the most common phage names are given. Phages present in strain 1 and 15 are presented in Benz et al. (2020).

Region	Length (kb)	Completeness (score)	Specific key words	Pos.	tRNA #	Total protein #	Phage hit protein #	Hyothetical hit protein #	Phage + hypothetical protein %	Bact. protein #	Attachment site	Phage species number *	Most common phage name**
1	129.4	Intact (150)	terminase, head, portal, tail, transposase	1-29488	0	31	29	1	96.70%	1	no	10	PHAGE_Entero_BP_4795_NC_004813(19)
2	49.5	Intact (150)	integrase, transposase	1788033-1816671	3	65	48	15	96.90%	2	yes	28	PHAGE_Pectob_ZF40_NC_019522(13)
9	44.8	Intact (150)	integrase, lysis, lysin, terminase, portal, protease, tail, plate	3879120-3923987	0	52	48	4	100%	0	yes	9	PHAGE_Entero_mEp460_NC_019716(37)
10	36.8	Intact (150)	recombinase, tail	4573693-4610496	0	55	46	9	100%	0	no	15	PHAGE_Burkho_BcepMu_NC_005882(31)
11	50.5	Intact (150)	integrase, lysin, tail	4822982-4873482	0	54	48	1	90.70%	5	yes	21	PHAGE_Entero_lambda_NC_001416(25)

*the number of different phages that have similar proteins to those in the region

**note that only one of many names is shown

Table S5: Primers used to generate knockout mutants of p1ESBL and p15ESBL.

Primer	Direction	Construct	Sequence (5’> 3’)
pFB119	fw	p1ESBL/ p15ESBL (mutA/mutB)	gtacctggaattttgaggacggtgccgggtactggatgaa-TGTAGGCTGGAGCTGCTTC
pFB121	rev	p1ESBL/ p15ESBL (mutA)	tgctgccgccccgtctccggcggggcggtgtggttgttca-TGAATATCCTCCTTAGTTCC
pFB122	rev	p1ESBL/ p15ESBL (mutB)	cgcatccacacgggaacctgatattgttcgtctgatttca-TGAATATCCTCCTTAGTTCC
pFB123	fw	Verification	ggagcagacggaatggcaccg
pFB124	rev	Verification	gtcacgggctgccattcgc

5) Supplementary Datasets

Dataset S1: Accessory genes of plasmid p1ESBL, generated with VrProfile (19). The following features were tested: **VF**; Virulence factor, **AR**; Antibiotic resistance determinant, **T3SE**; (TE) type III secretion effector, **T4SE**; (FE) type IV secretion effector, **T6SE**; (SE) type VI secretion effector, **T7SE**; type VII secretion effector, **Prophage**; Prophage, **ICE**; integrative and conjugative element, **T3SS**; (T) type III secretion system, **T4SS**; (F) type IV secretion system, **T6SS**; (S) type VI secretion system, **T7SS**; type VII secretion system, **Integron**; Integron, **IS**; IS element, **PAI**; Pathogenicity island, **ARI**; Resistance island.

Dataset S1 is provided in a separate file, Dataset_S1.xlsx

Dataset S2: Accessory genes of plasmid p15ESBL generated with VrProfile (19). The following features were tested: **VF**; Virulence factor, **AR**; Antibiotic resistance determinant, **T3SE**; (TE) type III secretion effector, **T4SE**; (FE) type IV secretion effector, **T6SE**; (SE) type VI secretion effector, **T7SE**; type VII secretion effector, **Prophage**; Prophage, **ICE**; integrative and conjugative element, **T3SS**; (T) type III secretion system, **T4SS**; (F) type IV secretion system, **T6SS**; (S) type VI secretion system, **T7SS**; type VII secretion system, **Integron**; Integron, **IS**; IS element, **PAI**; Pathogenicity island, **ARI**; Resistance island.

Dataset S2 is provided in a separate file, Dataset_S2.xlsx

Dataset S3: Description of genes in the leading region of p1ESBL and p15ESBL and their comparison to genes in the leading region of R64 (IncI1, accession AP005147) and pCT (IncK, accession FN868832). For genes indicated with a *, we performed functional predictions with Phyre^2 homology modeling (21).

Dataset S3 is provided in a separate file, Dataset_S3.xlsx

Dataset S4: Accessory genes within the region of the putative chromosomal PAI of strain 19 (contig 1; position 1’777’892-1’932’181) generated with VrProfile (19). The following features were tested: **VF**; Virulence factor, **AR**; Antibiotic resistance determinant, **T3SE**; (TE) type III secretion effector, **T4SE**; (FE) type IV secretion effector, **T6SE**; (SE) type VI secretion effector, **T7SE**; type VII secretion effector, **Prophage**; Prophage, **ICE**; integrative and conjugative element, **T3SS**; (T) type III secretion system, **T4SS**; (F) type IV secretion system, **T6SS**; (S) type VI secretion system, **T7SS**; type VII secretion system, **Integron**; Integron, **IS**; IS element, **PAI**; Pathogenicity island, **ARI**; Resistance island.

Dataset S4 is provided in a separate file, Dataset_S4.xlsx

Dataset S5: Accessory genes within the region of the putative chromosomal PAI of strain 1 (contig ESBL01_6; position 19’420-40’408) generated with VrProfile (19). The following features were tested: **VF**; Virulence factor, **AR**; Antibiotic resistance determinant, **T3SE**; (TE) type III secretion effector, **T4SE**; (FE) type IV secretion effector, **T6SE**; (SE) type VI secretion effector, **T7SE**; type VII secretion effector, **Prophage**; Prophage, **ICE**; integrative and conjugative element, **T3SS**; (T) type III secretion system, **T4SS**; (F) type IV secretion system, **T6SS**; (S) type VI secretion system, **T7SS**; type VII secretion system, **Integron**; Integron, **IS**; IS element, **PAI**; Pathogenicity island, **ARI**; Resistance island.

Dataset S5 is provided in a separate file, Dataset_S5.xlsx

Dataset S6: Comprehensive list of genetic changes identified comparing ancestral with evolved clones. Non-self-explanatory column names: Pop ID = Identifier of the population the clones were isolated from; Clone ID = Clone specific identifier (p+/p-); Col = Colour, Contig = Contig according to deposited sequences; Del start/ end = Start and stop of deletions larger than 10 bp.

Dataset S6 is provided in a separate file, Dataset_S6.xlsx

Dataset S7: Protein structure prediction for YfhA in the plasmid leading region. The top 15 structural homologies predicted with $Phyre^2$ (21) are shown.

Dataset S7 is provided in a separate file, Dataset_S7.xlsx

6) Supplementary References

1. O. Clermont, S. Bonacorsi, E. Bingen, Rapid and simple determination of the Escherichia coli phylogenetic group. *Appl. Environ. Microbiol.* **66**, 4555–4558 (2000).
2. O. Clermont, J. K. Christenson, E. Denamur, D. M. Gordon, The Clermont Escherichia coli phylo-typing method revisited: Improvement of specificity and detection of new phylo-groups. *Environ. Microbiol. Rep.* **5**, 58–65 (2013).
3. E. Bakkeren, *et al.*, Pathogen invasion-dependent tissue reservoirs and plasmid-encoded antibiotic degradation boost plasmid spread in the gut. *Elife* **10**, 1–32 (2021).
4. L. Hale, O. Lazos, A. S. Haines, C. M. Thomas, An efficient stress-free strategy to displace stable bacterial plasmids. *Biotechniques* **48**, 223–228 (2010).
5. M. Diard, *et al.*, Inflammation boosts bacteriophage transfer between Salmonella spp. *Science (80-.).* **355**, 1211–1215 (2017).
6. K. A. Datsenko, B. L. Wanner, One-step inactivation of chromosomal genes in Escherichia coli K-12 using PCR products. *Proc. Natl. Acad. Sci. U. S. A.* **97**, 6640–6645 (2000).
7. S. K. Sharan, L. C. Thomason, S. G. Kuznetsov, D. L. Court, Recombineering: A homologous recombination-based method of genetic engineering. *Nat. Protoc.* **4**, 206–223 (2009).
8. T. T. Hoang, R. R. Karkhoff-Schweizer, A. J. Kutchma, H. P. Schweizer, A broad-host-range Flp-FRT recombination system for site-specific excision of chromosomally-located DNA sequences: application for isolation of unmarked Pseudomonas aeruginosa mutants. *Gene* **212**, 77–86 (1998).
9. D. Qiu, F. H. Damron, T. Mima, H. P. Schweizer, H. D. Yu, PBAD-Based Shuttle Vectors for Functional Analysis of Toxic and Highly Regulated Genes in Pseudomonas and Burkholderia spp. and Other Bacteria. *Appl. Environ. Microbiol.* **74**, 7422 (2008).
10. A. M. Cavaleiro, S. H. Kim, S. Seppälä, M. T. Nielsen, M. H. H. Nørholm, Accurate DNA Assembly and Genome Engineering with Optimized Uracil Excision Cloning. *ACS Synth. Biol.* **4**, 1042–1046 (2015).
11. L. Simonsen, D. M. Gordon, F. M. Stewart, B. R. Levin, Estimating the rate of plasmid transfer: An end-point method. *J. Gen. Microbiol.* **136**, 2319–2325 (1990).
12. J. S. Huisman, *et al.*, Estimating plasmid conjugation rates: A new computational tool and a critical comparison of methods. *Plasmid* **121**, 102627 (2022).
13. L. De Gelder, J. M. Ponciano, P. Joyce, E. M. Top, Stability of a promiscuous plasmid in different hosts: no guarantee for a long-term relationship. *Microbiology* **153**, 452–463 (2007).
14. B. Headd, S. A. Bradford, The conjugation window in an Escherichia coli K-12 strain with an IncFII plasmid. *Appl. Environ. Microbiol.* **86** (2020).
15. L. S. Frost, J. Manchak, F- phenocopies: characterization of expression of the F transfer region in stationary phase. *Microbiology* **144 (Pt 9)**, 2579–2587 (1998).
16. J. E. Barrick, *et al.*, Identifying structural variation in haploid microbial genomes from short-read resequencing data using breseq. *BMC Genomics* **15**, 1–17 (2014).
17. T. Dimitriu, A. C. Matthews, A. Buckling, Increased copy number couples the evolution of plasmid horizontal transmission and plasmid-encoded antibiotic resistance. *Proc. Natl. Acad. Sci. U. S. A.* **118**, e2107818118 (2021).
18. F. Benz, *et al.*, Plasmid- and strain-specific factors drive variation in ESBL-plasmid spread in vitro and in vivo. *ISME J.* **15**, 862–878 (2020).
19. J. Li, *et al.*, VRprofile: gene-cluster-detection-based profiling of virulence and antibiotic resistance traits encoded within genome sequences of pathogenic bacteria.

Brief. Bioinform. **19**, 566–574 (2018).
20. T. Wein, *et al.*, Essential gene acquisition destabilizes plasmid inheritance. *PLoS Genet.* **17**, e1009656 (2021).
21. L. A. Kelley, S. Mezulis, C. M. Yates, M. N. Wass, M. J. E. Sternberg, The Phyre2 web portal for protein modeling, prediction and analysis. *Nat. Protoc.* **10**, 845–858 (2015).
22. D. Arndt, *et al.*, PHASTER: a better, faster version of the PHAST phage search tool. *Nucleic Acids Res.* **44**, W16–W21 (2016).
